# Interferon and immunity: the role of microRNA in viral evasion strategies

**DOI:** 10.3389/fimmu.2025.1567459

**Published:** 2025-05-09

**Authors:** Mobina Bayat, Rahil Nahid-Samiei, Javid Sadri Nahand, Behrouz Naghili

**Affiliations:** ^1^ Infectious and Tropical Diseases Research Center, Tabriz University of Medical Sciences, Tabriz, Iran; ^2^ Molecular Medicine Research Center, Tabriz University of Medical Sciences, Tabriz, Iran; ^3^ Department of Virology, School of Medicine, Iran University of Medical Sciences, Tehran, Iran

**Keywords:** microRNA, virus, interferon, innate immune response, viral-encoded miRNA

## Abstract

Interferons (IFNs) are indispensable innate antiviral cytokines that orchestrate the vertebrate immune response against viral incursions. Nearly every cell possesses the remarkable ability to release IFNs upon detecting viral threats, triggering a robust signaling cascade that alerts neighboring cells and halts viral propagation via paracrine communication. The intricate influence of IFNs is mediated by an extensive network of proteins activated through the Jak-STAT pathways, facilitating the swift transcription of over 300 interferon-stimulated genes (ISGs) that fortify cellular defenses against replication. However, the cunning nature of viruses has led to the evolution of sophisticated evasion strategies, notably through the manipulation of host microRNAs (miRNAs) that disrupt vital components of the IFN signaling machinery. This review delves into the intricate interplay between viral infections and both host- and viral-derived miRNAs, exploring their potent roles in modulating RIG-I-like receptors, Toll-like receptors, IFN receptors, and the JAK/STAT pathway, ultimately shaping the landscape of antiviral immunity.

## 1 Introduction

The innate immune system serves as the initial defense mechanism encountering viral infections. Host cells employ pattern recognition receptors (PRRs) to detect conserved structures associated with pathogens, which subsequently engage various adaptor proteins that facilitate downstream signaling and activate the interferon (IFN) response (1). The IFN system is universally found in vertebrates and plays a pivotal role in antiviral defense mechanisms (2). Interferons are categorized into three distinct families based on their receptor interactions, mode of induction, biological functions, and amino acid homology (3): type I, II, and III IFNs. Type I IFNs (IFN-I) were initially identified by their antiviral activity (4) and in mammals, IFN-I response is critical for innate antiviral responses (2).

**Figure 1:**
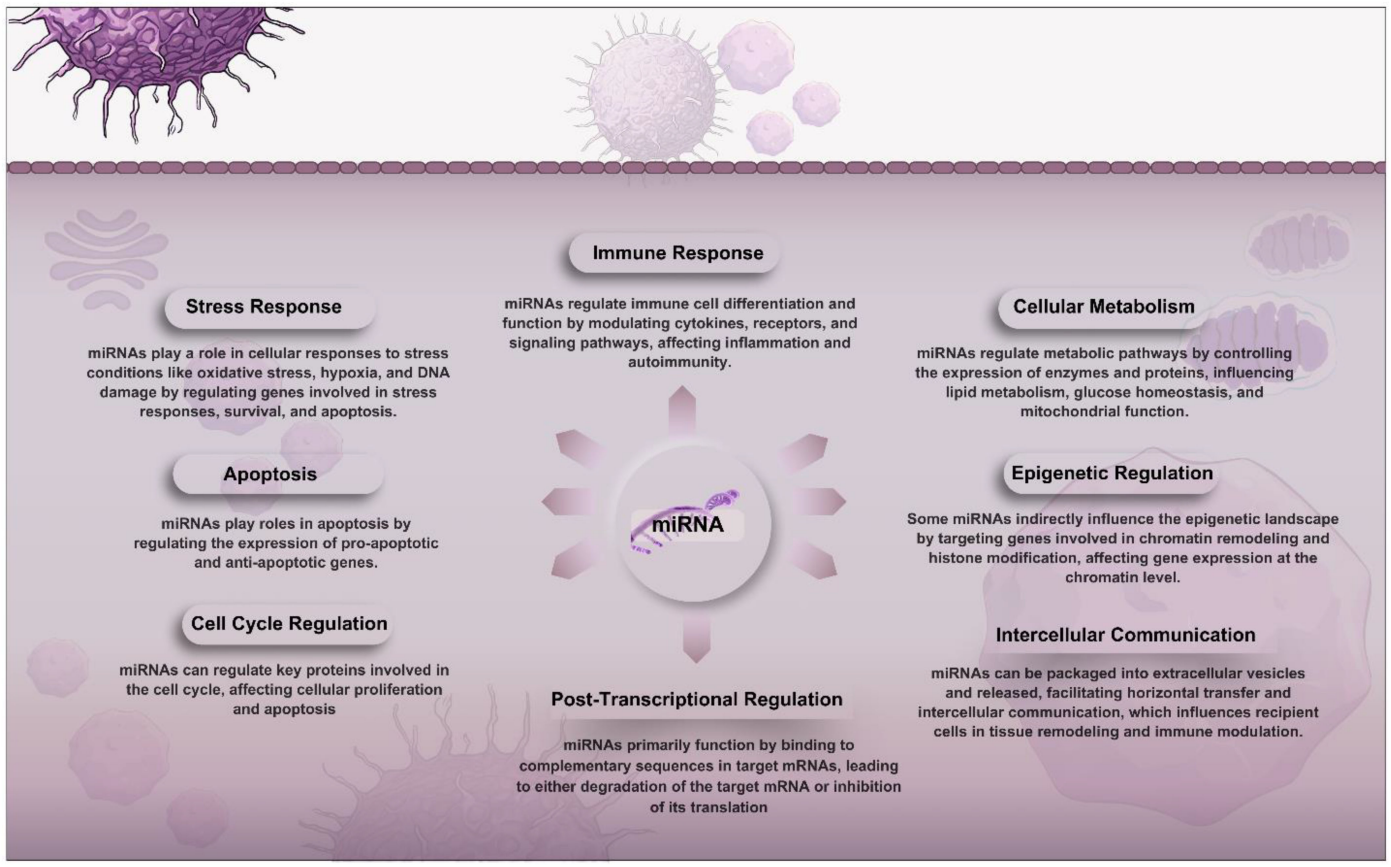
Overview of the roles of miRNAs in cellular functions. They function primarily through post-transcriptional regulation by binding to target mRNAs and guiding the RNA-induced silencing complex (RISC) for degradation or translation inhibition. miRNAs impact the cell cycle, apoptosis, stress responses, immune functions, metabolism, and epigenetic regulation. Additionally, they facilitate intercellular communication by being packaged into extracellular vesicles that influence recipient cells, contributing to tissue remodeling and immune modulation.

Viruses use several mechanisms to bypass the host IFN responses in order to replicate and continue their infectious cycle (4). One such mechanism is the deregulation of host microRNAs (miRNAs), which specifically target key factors involved in the IFN signaling pathway. This manipulation of miRNA expression levels allows viruses to disrupt the normal functioning of the IFN-mediated antiviral response, thereby gaining a selective advantage and ultimately contributing to their persistence within the host. MicroRNAs, characterized by their diminutive size and non-coding functionality, wield a significant influence on gene expression as key regulators. With an average length of approximately 22 nucleotides, these molecules exhibit a remarkable capacity for precision in their interactions with specific mRNA targets, binding to the 3’-untranslated regions (UTRs) and thereby precipitating either a diminution of mRNA translation or the degradation of the mRNA itself. This profound impact on gene expression serves to modulate the phenotypic outcomes of cellular processes (5–8). MicroRNAs, by virtue of their far-reaching influence on protein-coding genes, exert a profound impact on regulatory networks, including the host immune response (9, 10) (**Figure 1**). Notably, these non-coding RNAs have been confirmed to play a vital role in modulating the IFN cell signaling pathway, with numerous studies highlighting their contribution to this process (11–16). Intriguingly, viruses have evolved mechanisms to subvert the regulatory function of host miRNAs, while also encoding their own miRNAs to evade the host’s immune response. Notably, miRNAs exert fine-tuned control over the PRR-IFN signaling axis, where they can amplify or dampen antiviral signaling cascades to dynamically calibrate the host’s response to viral invasion (17). In fact, viruses have adapted the host transcriptional machinery to their advantage, enabling them to produce viral miRNAs that serve their own interests (18, 19). This review aims to elucidate how viruses manipulate the IFN signaling pathway by disrupting host miRNAs and deploying viral miRNAs. By shedding light on these mechanisms, we hope to provide insights into the development of more effective strategies for preventing and treating diverse viral infections.

## 2 Innate immune system

The immune system encompasses a diverse array of cells, chemicals, and processes that work synergistically to safeguard the skin, respiratory pathways, gastrointestinal tract, and other regions from foreign antigens, including microbes (such as bacteria, fungi, and parasites), viruses, neoplastic cells, and toxins. In addition to structural and chemical barriers that offer initial protection against infections, the immune system can be broadly conceptualized as comprising two primary “lines of defense”: innate immunity and adaptive immunity (10, 20, 21). The innate immune response constitutes the primary, frontline defense against invading pathogens, providing an antigen-independent, non-specific mechanism of defense that is promptly deployed by the host upon antigen encounter. This immediate and non-specific response enables the host to rapidly and effectively counteract the incursion of pathogens, thereby safeguarding against the establishment of infection (19, 22, 23).

The innate immune system serves as a universal and fundamental defense mechanism against infectious diseases, employing various recognition strategies to detect the presence of pathogens. A key mechanism involves pattern recognition receptors (PRRs), which identify conserved microbial structures that are characteristic of entire classes of microorganisms. These receptors are adept at recognizing metabolic byproducts unique to specific microbial classes, such as lipopolysaccharides found in Gram-negative bacteria (24).

In the case of viral pathogens, the absence of distinct molecular components in host cells shifts the focus of innate immune recognition to viral nucleic acids. The innate immune system has evolved to recognize specific structural features of viral RNA and DNA that differentiate them from host nucleic acids. This includes the detection of long double-stranded RNA (dsRNA), RNA with 5’-triphosphate groups, and unmethylated CpG motifs present in viral DNA genomes. However, recognizing these features alone is not enough to reliably differentiate between host and viral nucleic acids. Thus, the innate immune response relies on additional factors and context to accurately determine the origin of the nucleic acids it encounters [see (24)].

The mechanisms of innate immune recognition can be divided into two primary categories: cell-intrinsic and cell-extrinsic, based on whether the response is initiated by infected cells or by those that remain uninfected. Cell-intrinsic recognition occurs mainly within infected cells and is facilitated by cytosolic sensors such as NOD-like receptors (NLRs) and RIG-I-like receptors (RLRs). These receptors are expressed broadly, allowing for the detection of viral pathogens that can infect various cell types. In contrast, cell-extrinsic recognition involves transmembrane receptors, such as Toll-like receptors (TLRs) and C-type lectins (CLRs), which do not necessitate the infection of the cells expressing them. This form of recognition is predominantly carried out by specialized immune cells, including plasmacytoid dendritic cells, macrophages, and conventional dendritic cells, which are essential for initiating and coordinating the immune response (24).

The viral recognition by the innate immune system leads to two primary outcomes. First, it triggers the innate antiviral mechanisms, which are largely mediated by type-I interferons (IFNs). Second, it activates the adaptive immune response, which offers a more targeted, antigen-specific, and long-lasting antiviral immunity. An essential tactic in the host’s defense against viral infections is the removal of cells that have been infected by the virus (24, 25). This elimination may occur through intrinsic mechanisms driven by IFN-I within the infected cells or through the activation of cytotoxic lymphocytes, particularly natural killer (NK) cells and CD8+ T cells.

## 3 Interferons and their regulation by miRNAs during viral infections

Antiviral mechanisms in vertebrates are fundamentally dependent on the activity of IFNs. IFNs constitute a family of cytokines that play a pivotal role early in the innate immune response, possessing the capacity to induce an antiviral state in both infected and uninfected neighboring cells (26). In addition to their antiviral functions, interferon cytokines are also integral to the regulation of the ensuing adaptive immune response, thereby facilitating a coordinated defense against viral pathogens (25).

The IFN family is categorized into three distinct types. The type I IFN family includes a multi-gene cytokine group, with 13 partially homologous IFN-α subtypes identified in humans and 14 in mice, in addition to a single IFN-β and several less well-characterized gene products (27, 28). In contrast, the type II IFN (IFN-II) family consists of a singular gene product, IFN-γ, predominantly produced by T cells and natural killer (NK) cells. This cytokine interacts with a broad spectrum of cell types expressing the IFN-γ receptor (29). The type III IFN (IFN-III) family includes IFN-λ1, IFN-λ2, and IFN-λ3 (also designated as IL-29, IL-28A, and IL-28B), as well as the recently identified IFN-λ4. These cytokines share functional similarities with those of the type I IFN family; however, their activity is more limited due to the restricted expression of their receptor on epithelial cell surfaces (30, 31). It is noteworthy that immune cells tend to exhibit minimal responsiveness to IFN-λ (32–34).

IFN-α and IFN-β are well-recognized for their capacity to establish an antiviral environment in both infected and unaffected bystander cells. They accomplish this by initiating a gene transcription program that interferes with various stages of the viral replication process through several mechanisms (35). Beyond their antiviral properties, these cytokines play a significant role in modulating both innate and adaptive immune responses, not only against viral pathogens but also against bacterial and other infectious agents.

**Figure 2:**
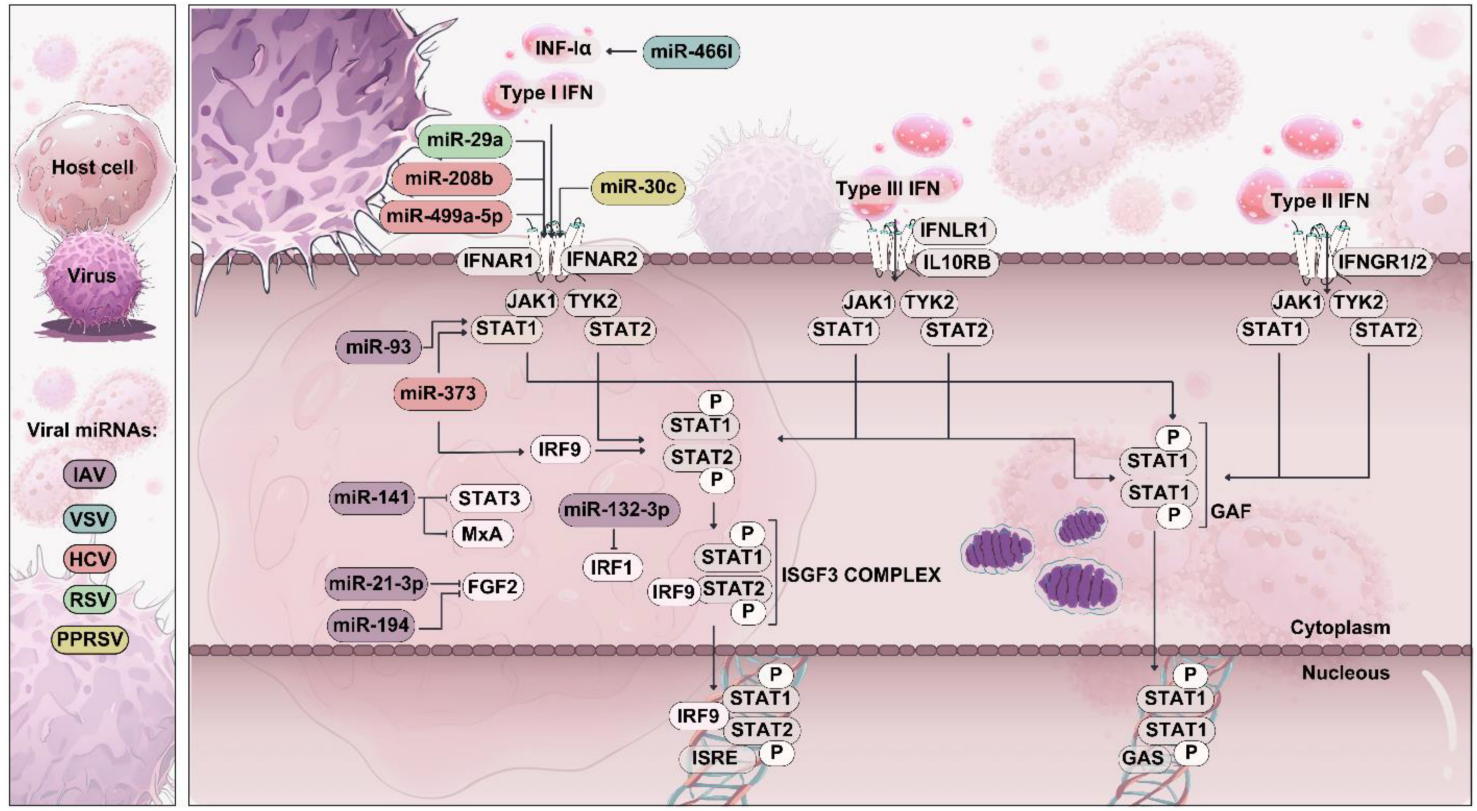
Type I, type II, and type III IFN signaling pathways. Type I and Type III IFNs bind to distinct receptor complexes, triggering the activation of downstream signaling pathways. IFN-I (e.g., IFN-α/β) bind to the IFNAR1/IFNAR2 receptor complex, leading to the phosphorylation of STAT1 and STAT2, which form a trimeric complex called ISGF3. ISGF3 then translocate to the nucleus, binding ISREs and inducing the transcription of various ISGs. Viral miRNAs can target molecules in the IFN signaling pathways, leading to the inhibition of IFN production and reduced antiviral immunity.

The effectiveness of the IFN response during infectious diseases is highly context-dependent, shaped by factors such as the specific conditions arising from the infection, the timing and site of IFN signal delivery, and the downstream signaling pathways activated by the IFN-I receptor. These variables can result in either beneficial or adverse effects for the host (24) (**Figure 2**).

Given the critical role of IFNs in antiviral defense, their regulation by miRNAs during viral infections is a key focus of this section. miRNAs fine-tune gene expression, including components of the IFN pathway, either enhancing or suppressing the antiviral response. To improve clarity, this section is structured around the stages of the IFN response modulated by miRNAs, from induction to effector functions and negative regulation.

### 3.1 Activation of interferons

The activation of various PRRs such as TLR3, TLR4, TLR7, TLR8, TLR9, and the RIG-I-like receptors (RLRs) such as RIG-I and MDA-5, along with cytosolic DNA sensors, culminates in the production of IFN-I. Typically, virally infected cells activate the cell-intrinsic pathway for IFN-I production through the engagement of RLRs or DNA sensors. In contrast, plasmacytoid dendritic cells (pDCs) are capable of detecting viral genomes within endosomes via the activation of TLR7 or TLR9, which leads to a robust and sustained release of IFN-α and IFN-β (24, 36). pDCs are characterized by their ability to maintain elevated basal levels of interferon regulatory factor 7 (IRF7). They possess a unique capability to directly connect TLR signaling to IRF7, thereby facilitating a rapid and intense transcriptional activation of IFN-I genes. This mechanism underscores their critical role in the early innate immune response to viral infections (24).

This activation sets the stage for IFN production, which is tightly regulated by miRNAs, as explored in the following subsections.

### 3.2 miRNAs modulating the induction of interferons during viral infections

miRNAs play a crucial role in modulating the initial induction of IFNs by targeting PRRs and their downstream signaling molecules. This regulation can either inhibit or enhance IFN production, influencing the host’s ability to mount an effective antiviral response.

#### 3.2.1 Targeting RIG-I-like receptors

RLRs, or retinoic acid-inducible gene I-like receptors, are a group of cytosolic RNA sensors that includes three key members: RIG-I, melanoma differentiation-associated protein 5 (MDA5), and laboratory of genetics and physiology 2 (LGP2). All three proteins share a similar structural design, featuring a central helicase domain and a carboxy-terminal domain (CTD) that collaboratively recognize immunostimulatory RNAs. RIG-I is unique in possessing an additional repressor domain (RD) at its C-terminus, which serves to inhibit its activation when in a resting state. Upon binding to viral RNA, RIG-I undergoes a conformational alteration that uncovers its caspase activation and recruitment domain (CARD), thereby triggering antiviral signaling pathways (37, 38). RIG-I functions as a vital pattern recognition receptor (PRR) with the ability to detect a diverse array of viruses, such as Sendai virus (SeV), vesicular stomatitis virus (VSV), influenza virus, hepatitis C virus (HCV), Japanese encephalitis virus (JEV), and Epstein-Barr virus (EBV). Notably, EBV has a small RNA genome encoded by its DNA. This extensive recognition capacity underscores RIG-I’s important role in the antiviral immune response, enabling it to effectively respond to various viral pathogens. By recognizing viral RNA, RIG-I initiates signaling pathways that lead to the production of type I interferons and other cytokines, ultimately enhancing the host’s defense mechanisms against invading viruses (24, 39).

MDA5 functions as a key sentinel for the detection of picornaviruses and can be activated by cytosolic synthetic dsRNA, including polyinosinic-polycytidylic acid (poly I:C) (39). There is notable redundancy in the recognition capabilities of MDA5 and RIG-I, as both sensors can induce type I interferon production in response to infections by viruses including dengue virus and West Nile virus. However, MDA5 demonstrates a preference for longer RNA molecules, specifically those exceeding 2 kilobases in length, while RIG-I is activated by a more diverse range of dsRNA sizes, spanning 400 base pairs to 4 kilobases (39, 40). This differential sensing mechanism enables the immune system to effectively respond to a variety of viral threats, ensuring robust antiviral signaling and the subsequent activation of innate immune responses.

miRNAs regulate RLR activity during viral infection as follows:

The discovery of RIG-I and MDA5 as inducers of type I interferons has represented a significant advancement in immunology over the past two decades (41, 42). Both RIG-I and MDA5, members of the RLR family, play essential roles in the recruitment of IPS-1 (known as Cardif, VISA, or MAVS), a multifunctional adaptor protein located on the outer mitochondrial membrane. This recruitment is a critical step that triggers a signaling cascade, activating key transcription factors such as IRF3, IRF7, and NF-κB. The activation of these transcription factors results in the expression of a broad array of genes, particularly those encoding IFNs-I. IFNs-I are pivotal in modulating immune responses and exerting antiviral effects, as they enhance the antiviral state of neighboring cells, promote the activation of immune cells, and inhibit viral replication. This interplay highlights the important role of RLRs and IPS-1 in orchestrating the host’s antiviral defense mechanisms (43). The intricate antiviral signaling mechanisms are tightly regulated through the activation of RIG-I and other key intermediates within the pathway. Post-translational modifications, particularly ubiquitylation and phosphorylation, have been identified as essential mechanisms that activate RIG-I, helping to enhance its function. Notably, many viruses have evolved strategies to target these modifications, aiming to delay or evade the host immune response. By disrupting these critical signaling pathways, viruses attempt to circumvent the host’s defenses, thereby promoting their own survival and replication (44, 45).

Rhabdovirus has been shown to induce the overexpression of miR-3570, which promotes viral replication by suppressing the production of IFNs and inhibiting the activation of the NF-κB and IRF3 signaling pathways, primarily through the downregulation of MAVS (46). Furthermore, the expression of miR-485 is induced in response to infections with various RNA viruses, including Newcastle disease virus (NDV) and influenza virus, as well as during the transfection of cells with poly(I:C), a synthetic mimic of viral dsRNA. Elevated levels of miR-485 have also been observed in human peripheral blood mononuclear cells (PBMCs) following such transfections. Importantly, miR-485 directly targets the 3’ UTR of the RIG-I gene, which plays a pivotal role in the recognition of viral RNA and the initiation of the antiviral immune response. Ectopic expression of miR-485 in both human and mouse cells has been shown to disrupt the RIG-I-dependent antiviral pathway, resulting in compromised antiviral signaling and increased viral loads. This highlights the critical role of miR-485 as a regulatory molecule that can modulate the host’s antiviral response, showcasing how RNA viruses may employ host cellular mechanisms, such as miRNAs, to evade immune detection and promote their replication. Through the regulation of key components like RIG-I, miRNAs such as miR-485 serve as important fine-tuning agents in the host’s immune response to viral infections. Ingle et al. (11) demonstrated that miR-485 forms a stable complex with human Argonaute 2 (Ago2) protein and is recruited to the RIG-I transcript during viral infections. This interaction suggests a significant role for miR-485 in the post-transcriptional regulation of RIG-I, a key sensor of viral RNA and an important player in the antiviral immune response. Additionally, miR-485 impacts the signaling cascade downstream of RIG-I, influencing the expression of genes encoding type I and III IFNs.

Overexpression of miR-485 has been shown to significantly inhibit the transcriptional activities of promoters for IFN-α4, IFN-β, ISRE (interferon-stimulated response element), and IL-29 in response to infections with NDV. Notably, infections with highly pathogenic viruses such as H5N1 and NDV have been observed to reduce the levels of IFN-α, IFN-β, IP10, and IL-29 mRNAs in the presence of miR-485 in both human and mouse cell lines.

Mechanistically, the upregulation of miR-485 by NDV and influenza A virus (IAV) facilitates viral replication by suppressing virus-induced expression of IFN-I and IFN-III through direct targeting of RIG-I. This emphasizes the sophisticated interplay between viral pathogens and host miRNA-mediated regulation. It highlights how viruses can manipulate host cellular pathways, particularly miRNA expression, to evade the immune response. Overall, these findings underscore the potential of miRNAs as critical modulators of antiviral immunity, presenting new avenues for understanding host-pathogen interactions and developing therapeutic strategies against viral infections (11).

The intricate dynamics between microRNAs and the immune system are exemplified by the dual roles of miR-92a and miR-218 in facilitating VSV replication. By targeting RIG-I in macrophages, these microRNAs inhibit the production of type I interferons, thereby creating an environment conducive to viral propagation (47, 48).

In a separate context, HBV-induced miR146a exhibits a distinct mechanism of action, suppressing the expression of both RIG-I and its enhancer RIG-G, leading to a diminution of IFN-I production and impairment of innate immunity (49). Furthermore, the studies by Lu et al. (50) highlight the intricate relationship between microRNAs and the immune system, particularly in the context of viral infections. Their research demonstrates that EBV-encoded miR-BART6-3p plays a crucial role in inhibiting genes involved in the RLR signaling pathway and the IFN-I response. Notably, this specific microRNA has a preferential effect in suppressing RIG-I-like receptor signaling-mediated production of IFN-β, highlighting its unique function in modulating the immune response during EBV infection (50). In contrast, miR-136 exhibits a protective effect on host defense against H5N1 IAV replication *in vitro*. Through its mechanism of action as a ligand for RIG-I during infection, miR-136 triggers innate immunity and IFN production, thereby bolstering the host’s defense against viral invasion (51).

Fibroblast growth factor 2 (FGF2), a multifaceted signaling molecule, has been implicated in a diverse array of biological processes, such as the intricate dynamics of angiogenesis, the complex cascade of embryonic development, and the orchestrated response of wound healing (52). Furthermore, recent studies have suggested an unexpected role for FGF2 in innate immune signaling, where it interacts with the RIG-I receptor family members MDA5 and RIG-I (53). Although FGF2 has been shown to bind to both receptors, the majority of research has focused on its interaction with RIG-I, a dsRNA helicase that plays a crucial role in the recognition of viral RNA (37). The binding of FGF2 to RIG-I’s CARD domain stabilizes the protein by preventing its ubiquitination and subsequent proteasomal degradation (53). Surprisingly, FGF2 has been found to negatively regulate RIG-I functioning by disrupting IPS-1 binding suggesting a complex interplay between FGF2 and RIG-I. This dichotomous behavior highlights the intricate nuances of FGF2’s role in innate immunity (53). In a striking contrast to its negative regulatory effects on RIG-I, administration of recombinant FGF2 protein has been shown to significantly alleviate the severity of IAV-induced lung injury and promote the survival of IAV-infected animals by recruiting neutrophils. These results indicate that FGF2 can play a vital role in modulating the host’s response to viral infection, underscoring the importance of further investigation into its immunomodulatory functions (54).

**Figure 3:**
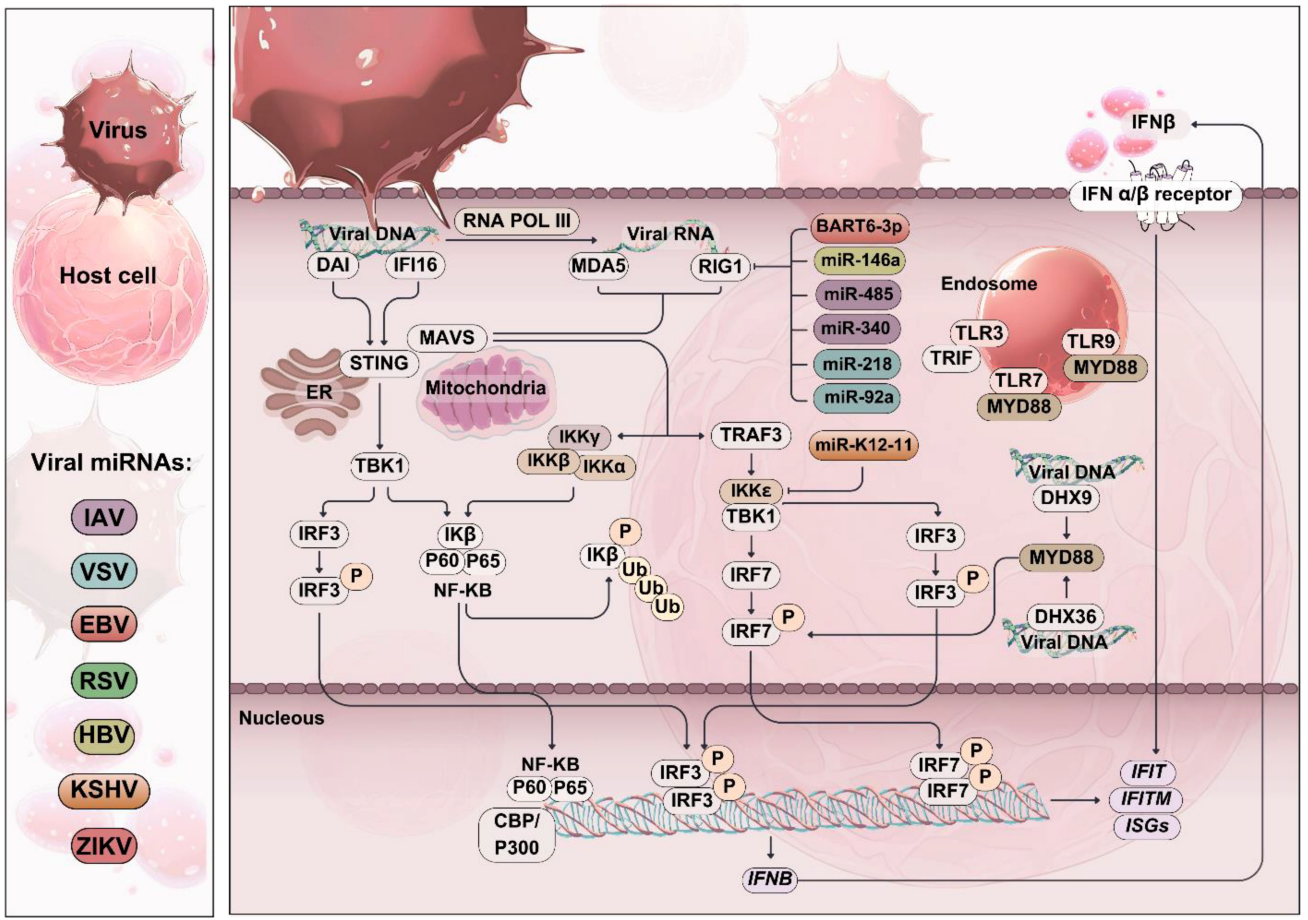
Role of RIG-I-like receptors (RLRs) in viral infection. Schematic representation of viral invasion and RIG-I-like receptor (RLR) activation. Viral replication in the host cell produces pathogen-associated molecular patterns (PAMPs) such as viral dsRNA, which is recognized by RLRs. Upon activation, RLRs induce the expression of type I interferons (IFNs) and pro-inflammatory cytokines through the activation of downstream signaling cascades, including IRF-3/7 and NF-κB. Viral miRNAs can target and inhibit the activation of RLRs and their downstream signaling cascades, leading to decreased expression of IFN-β and other pro-inflammatory cytokines. This diminishes the host’s antiviral response and promotes viral evasion of host immunity.

A study published by Wang et al. (55) has highlighted the complex interaction between FGF2 isoforms and the immune response during IAV infection. They demonstrated that overexpressing high-molecular-weight isoforms of FGF2 in A549 cells results in enhanced production of IFN-I and suppressed viral replication in response to IAV challenge. This suggests that different molecular weight isoforms of FGF2 may have unique functions in modulating antiviral responses (55). Importantly, the study revealed that miR-194 directly targets FGF2, leading to decreased expression of FGF2 at both mRNA and protein levels. The upregulation of miR-194 was shown to facilitate IAV replication by downregulating IFN-I production. Conversely, reintroducing FGF2 could counteract these effects, demonstrating that FGF2 plays a vital role in the antiviral response. Inhibition of miR-194 was found to mitigate IAV-induced lung injury *in vivo*, likely due to enhanced IFN-I antiviral activities. Further investigation uncovered that FGF2 activates the RIG-I signaling cascade, while miR-194 suppresses the phosphorylation of tank-binding kinase 1 (TBK1) and IFN regulatory factor 3 (IRF3) (55). Overall, the study suggests that miR-194 downregulates IFN-I production triggered by IAV infection by targeting FGF2, thereby repressing the RIG-I pathway. Notably, FGF2 can block the miR-194-induced decrease in IFN-I, reinforcing its role in activating RIG-I signaling. In a related study, it was also found that miR-21-3p promotes IAV (H5N1) replication through downregulation of IFN-I response via targeting FGF2 (56) (**Figure 3**). This further underscores the role of specific miRNAs in manipulating host immune responses, particularly in the context of viral infections, and illustrates the potential for targeting these pathways in therapeutic strategies against such pathogens.

Under normal physiological conditions, the RIG-I/MDA5 pathway is latent, existing in a state of repression within the cytoplasm (57, 58). However, upon viral infection, RIG-I/MDA5 undergoes a paradigmatic shift, as it recognizes viral RNA and undergoes conformational changes that enable its activation. These alterations promotes the interaction between RIG-I/MDA5 and MAVS, a key adaptor protein localized to the surface of mitochondria (59). The MAVS-mediated dimerization event initiates a signaling cascade that serves as a hub for complex assembly at the outer mitochondrial membrane. This platform attracts key molecules, including TRAF6, which is recruited to the IRAK1-IRAK4-MyD88 adaptor complex. Upon recruitment, TRAF6 undergoes K63-linked polyubiquitination, thereby creating a binding site for downstream regulators. Specifically, TAK1 and TAB2/3 proteins bind to this ubiquitinated TRAF6, triggering the activation of the TAK1-TAB2/3 complexes. This activation subsequently results in the nuclear translocation of NF-κB, ultimately driving the transcriptional activation of genes involved in producing the inflammatory cytokines (60, 61).

The RLRs interact with MAVS, initiating a signaling cascade that recruits various proteins, including TRAF3, TBK1, IKKϵ, IKKγ (also known as NEMO), IKKα, and IKKβ. This recruitment culminates in the activation and nuclear translocation of the transcription factors IRF3 and NF-κB, which play pivotal roles in the antiviral response (62, 63). Research has shown that miRNAs can inhibit the interferon response and suppress the innate immune system during viral infections by regulating the expression of key factors. For instance, miR-200b-3p plays a role in the replication of IAV and VSV by suppressing NF-κB activity and promoting IFN-I production via IRF3-mediated signaling, which is achieved through targeting TBK1 (64). Similarly, when SCRV is present, miR-15b promotes viral replication by suppressing the production of IFN-I and inflammatory cytokines through the inhibition of TBK1 (65, 66). Liang et al. discovered that IKKε is directly targeted by the microRNA miR-K12-11 encoded by Kaposi’s sarcoma-associated herpesvirus (KSHV). Their investigation revealed that increased levels of miR-K12-11 led to downregulation of IKKε, whereas inhibiting miR-K12-11 restored IKKε levels in cells infected with KSHV. Additionally, the authors established that the expression of miR-K12-11 disrupts interferon signaling pathways, which in turn diminishes antiviral immune responses (67). These results illustrate the capacity of viral microRNAs to modulate immune signaling and promote the viral lifecycle. Given that the innate immune response is linked to the reactivation of KSHV, and considering that miR-K12-11 is expressed during both the latent and lytic stages of infection, it is proposed that miR-K12-11 may play a role in preserving KSHV latency by targeting IKKε (67).

#### 3.2.2 Targeting toll-like receptors during viral infections

TLRs represent the most extensively researched components of the PRR family, serving to identify conserved molecular signatures present across a diverse array of microorganisms. In mice, there are 11 functional TLRs (TLR1-7, 9, 11-13), whereas humans have TLR1-10. All TLRs are characterized as type I transmembrane proteins, featuring leucine-rich repeat (LRR) domains within their ectodomains that facilitate the recognition of PAMPs. Certain TLRs are adept at recognizing bacterial, fungal, and protozoan entities, while others are specifically tailored to identify viral pathogens (68, 69).

Upon the detection of a virus, the activation of TLRs initiates a multifaceted signaling cascade that diverges into two primary pathways. This process ultimately culminates in the secretion of pro-inflammatory cytokines through the NF-κB pathway and the production of antiviral cytokines, specifically IFN-I, via the IRF pathway. Moreover, TLR signaling is known to engage mitogen-activated protein kinases (MAPKs). Specific TLRs, including TLR3, TLR7, TLR8, and TLR9, located within the endosomal membrane, are responsible for discriminating viral pathogens. These receptors can interact with viral nucleic acids following the endocytosis of viral particles (69). The endosomal TLRs, specifically TLR3/7/9 depend on the endoplasmic reticulum membrane protein UNC93B for their trafficking to the endosome. UNC93B forms a physical interaction with these TLRs via their transmembrane domains, facilitating their transport to the endosomal compartment. Upon arrival in the endosome, the TLRs associate with adaptor proteins, namely TRIF and MYD88, which activate interferon regulatory factors IRF3 and IRF7. Once activated, IRF3 and IRF7 bind to the promoter region of the IFN-β gene, triggering its transcription. The consequently released IFN-β initiates paracrine signaling through the IFNα/β receptor, which in turn activates a myriad of interferon-stimulated genes (ISGs). Additionally, phosphorylated IRF3 has the capability to independently activate specific ISGs, including those encoding IFIT and IFITM genes, without reliance on IFN signaling (24, 70).

During infections caused by viruses or bacteria, as well as during the phagocytosis of apoptotic cells, DNA can be released and found within the cytoplasm and endosomes. Within the endosomal compartment, TLR9 plays a crucial role in recognizing unmethylated CpG DNA, which activates the adaptor protein MYD88. This activation leads to a downstream signaling cascade that promotes pro-inflammatory responses. Conversely, DNA that is present in the cytoplasm can be recognized by other sensors, such as DAI (DNA-dependent activator of interferon-regulatory factors) or IFI16 (interferon gamma-inducible protein 16). This recognition triggers a distinct signaling pathway that is dependent on the activation of STING (Stimulator of Interferon Genes), leading to the activation of both IRF3 and NF-κB. Furthermore, the activated cytoplasmic DNA is transcribed by RNA polymerase III into short RNA molecules that possess a 5’-triphosphate (5’-ppp) motif. These short RNAs then serve as ligands for the cytosolic receptor RIG-I, triggering antiviral signaling pathways. In addition, helicases such as DHX9 and DHX36 can bind to specific DNA ligands, including CpG-A and CpG-B, within the cytosol. This interaction further induces MYD88 and IRF7-dependent signaling responses, enhancing the host’s immune reaction against the infection. This multilayered detection and response system ensures that the immune system effectively recognizes and responds to different forms of pathogenic DNA (24, 62).

miRNAs modulate TLR signaling during viral infections as follows:

Various viruses have evolved strategies to evade IFN-I responses by targeting TLR-dependent signaling pathways. They also manipulate host and viral miRNAs that regulate these pathways. For instance, during HCV infection, increased levels of miR-758 suppress the expression of TLR3 and TLR7. This suppression leads to reduced production of IFN-α and IFN-β, weakening the antiviral response. Such tactics highlight the complex interaction between viral evasion mechanisms and host immune regulatory pathways, which is crucial for developing effective antiviral therapies and vaccines (14). MiR-155 facilitates HIV replication by suppressing the antiviral innate immune response. It targets TLR3, NF-κB, and IRF-3, inhibiting key immune signaling pathways and diminishing the host’s ability to combat the virus. This interplay between miR-155 and HIV underscores potential therapeutic targets (71). The overexpression of let-7a/b in cells infected with JEV exacerbates neuroinflammation and neurodegeneration. This effect is mediated by an increase in TNF-α production and apoptosis, driven by the activation of TLR7 and the Notch signaling pathway (72). MiR-125b attenuates the innate immune response to HCV infection by downregulating the TLR2/MyD88 signaling pathway. Similarly, miR-140-5p enhances the replication of RSV by decreasing levels of IFNα and inflammatory factors through the inhibition of TLR4 (73) (**Table 1**).

**Table 1:** Effect of viral-deregulated cellular miRNAs on the TLR and RLR pathway.

Viral infection	MicroRNA (Up/Down)	Target	Note	Ref
*Retroviridae*				
HIV	miR-155	TLR3, NF-κB and IRF-3	miR-155 promotes HIV replication in HIV progresses by inhibiting anti-viral innate response through targeting TLR3, NF-κB and IRF-3.	(71)
*Flaviviridae*				
JEV	miR-155	–	miR-155 alleviates peripheral nerve injury induced by negatively regulating NF-κB regulated downstream genes.	(74)
JEV	let-7a/b	TLR7	JEV-overexpressed let-7a/b enhances neuro-inflammation and neurodegeneration by positively regulating TNF-α and apoptosis through targeting TLR7 and NOTCH pathway.	(72)
JEV	miR-29b	TNFIAP3	miR-29b contributes to JEV pathogenesis by promoting pro-inflammatory cytokines production through targeting TNFIAP3 in microglia cells.	(75)
JEV	miR-19b-3p	RNF11	JEV-induced miR-19b-3p promotes JEV-mediates inflammatory cytokine by activating NF-kB signaling through targeting RNF11.	(76)
HCV	miR-125b	TLR2/MyD88 signaling	miR-125b inhibits HCV-induced innate immune responses by negatively regulating TLR2/MyD88 signaling	(77)
ZIKV	miR-146a	TRAF6 and STAT-1	ZIKV NS1-induced miR-146a facilitates viral replication by suppressing pro-inflammatory through negatively regulating TRAF6 and STAT-1.	(78)
*Hepadnaviridae*				
HBV	miR-30e	TRIM38, TANK, ATG12, BECN1, SOCS3, and SOCS1	Upregulated miR-30e suppresses viral replication by enhancing innate immunity by targeting TRIM38, TANK, ATG12, BECN1, SOCS3, and SOCS1.	(79)
*Paramyxoviridae*				
RSV	miR−140−5p	TLR4	miR-140-5p contributes to RSV replication by negatively regulating the levels of IFNα and inflammation factors through targeting TLR4.	(73)
NDV	miR-30e	TRIM38, TANK, ATG12, BECN1, SOCS3, and SOCS1	Upregulated miR-30e enhances antiviral innate immunity by suppressing immune regulators (SOCS1, SOCS3, TRIM38, TANK) and autophagy-related genes (ATG12, BECN1), thereby restricting viral replication.	(79)
*Rhabdoviridae*				
VSV	miR-33/33*	MAVS	miR-33/33* negatively regulates innate anti-viral responses by targeting MAVS.	(80)
VSV	miR-136	–	Innate antiviral responses are enhanced by miR-136 through activation of RIG-I signaling	(51)
*Orthomyxoviridae*				
IAV	miR-125a or -b	MAVS	miR-125a or -b contributes to IAV replication by positively regulating inflammatory cytokines and negatively regulating antiviral cytokines through targeting A20 and MAVS, respectively.	(81)
H5N1	miR-136	–	miR-136 enhances innate anti-viral responses by activating RIG-I signaling.	(51)
H5N1	H5N1 miR-HA-3p	PCBP2	H5N1-encoded miRNA-like small RNA, miR-HA-3p, triggers cytokine storm by promoting proinflammatory cytokine production through targeting PCBP2.	(82)
IVA (H1N1)	miR-29c	–	IAV-overexpressed miR-29c negatively regulates NF-κB activity and the expression of proinflammatory and antiviral cytokines through increasing A20 levels.	(83)
Togaviridae				
CHIKV	miR-146a	TRAF6, IRAK1 and IRAK2	CHIKV-overexpressed miR-146a alleviates pro-inflammatory immune responses by negatively regulating NF-kB through decreasing TRAF6, IRAK1 and IRAK2 levels.	(84)
*Arteriviridae*				
PRRSV	miR-125b	NF-kB	miR-125b restricts PRRSV replication by negatively regulating NF-kB.	(85)
*Coronaviridae*				
OC43	miR-9	NFKB1	N protein of HCoV OC43 control the host inflammatory response by promoting NF-kB1 expression levels through inhibiting miR-9.	(86)
*Picornaviridae*				
EV71	miR-302 cluster	KPNA2	Upregulated miR-302 cluster inhibits inflammatory cytokine production by targeting KPNA2, in return, EV71 promotes inflammatory cytokine production by suppressing miR-302 family.	(87)
Herpesviridae				
KSHV	KSHV- miR-K9	IRAK1 and MYD88	KSHV-encoded miR-K9 regulates KSHV reactivation by regulating TLR7/8 signaling through targeting IRAK1 and MYD88.	(88)
HCMV	HCMV- miR-UL112-3p	TLR2	HCMV-encoded miR-UL112-3p modulates innate immune response by negatively regulating TLR2-dependent activation of NF-κB and IRAK1.	(89)
EBV	EBV BHRF1-2-5p	IL-1 receptor 1 (IL1R1)	EBV miR-BHRF1-2-5p inhibits inflammatory cytokine expression by targeting IL1R1.	(90)

Furthermore, viral-encoded microRNAs have the capacity to modulate the host’s innate immune response by TLRs. Specifically, human cytomegalovirus (HCMV)-encoded miR-UL112-3p exerts its effect by inhibiting TLR2-mediated activation of both NF-κB and IRAK1 (89). KSHV-encoded miR-K9 plays a role in controlling KSHV reactivation by modulating TLR7/8 signaling (88). HIV-derived miR-88 and miR-99, bind to TLR8 and stimulate the release of pro-inflammatory factors such as TNFα, IL-6, and IL-12 from macrophages. This mechanism may play a role in the chronic abnormal immune activation observed in HIV infection (91).

#### 3.2.3 Targeting signaling molecules

##### 3.2.3.1 Downstream signaling molecules are also miRNA targets

IRAK1 has been identified as a negative regulator of RIG-I-mediated IFN-I signaling, exerting a suppressive influence on downstream antiviral responses (92). In contrast, TRAF6 interacts with phosphorylated IRAK1 to facilitate the activation of NF-κB, which is crucial for the subsequent induction of type I IFN (93, 94). Dysregulation of the RIG-I pathway and IFN production, particularly through the targeting of MAVS and the TRAF6/IRAK1 complex by aberrant microRNA regulation, has been a subject of investigations in various studies (**Supplementary Table 1**). For instance, a study by Mo et al. confirmed that the overexpression of miR-146a-5p during hepatitis A virus (HAV) infection impairs RIG-I/MDA5-mediated IFN-I signaling by promoting the cleavage of TRAF6, a vital adaptor protein in the RIG-I/MDA5-mediated IFN-I signaling cascade (95). Furthermore, miR-146a has been shown to negatively regulate IFN-I production triggered by vesicular stomatitis virus (VSV) infection in macrophages, thereby enhancing VSV replication through its targeting of TRAF6 and IRAK1/2 (96). Additionally, IRAK1 and IRAK2 are involved in IFN-I production induced by VSV infection, interacting with Fas-associated death domain protein (FADD), an important adaptor molecule in the RIG-I signaling pathway, in a virus-induced manner (96). Both miR-146a and miR-125a, which are upregulated during viral infections, function as negative regulators of the RIG-I-dependent antiviral pathway by targeting key components such as TRAF6, IRAK1, and IRAK2. Specifically, miR-146a targets TRAF6, IRAK1, and IRAK2 to suppress the pathway, while miR-125a targets MAVS and TRAF6 to disrupt IFN-I signaling, ultimately promoting viral replication, including that of HCV (97). Moreover, microRNA-146a has been highlighted as playing a crucial role in enabling viruses to evade the antiviral response by targeting TRAF6 and IRAK2, particularly noted during enterovirus 71 (EV71) infection (98, 99). This underscores the significance of miR-146a and the associated regulatory pathways in modulating the innate immune response, illustrating how viruses can exploit host cellular mechanisms to enhance their replication and survival.

Importantly, miRNAs have been evidenced to directly target IFN mRNAs during viral infections. For example, Li et al. (13) established that miR-466l directly binds to the 3’ UTRs of various IFN-α mRNA species, particularly to the AU-rich elements (AREs) in the 3’ UTRs of multiple IFN-α subtypes. This interaction inhibits IFN-α production and promotes viral replication (13). Additionally, members of the miR-548 family have been shown to target the 3’ UTR of IFN-λ1 mRNA. Additional research has shown that miR-548 mimics reduce the expression of IFN-λ1, whereas complementary inhibitors can increase IFN-λ1 levels and induce the expression of ISGs. Significantly, miR-548 mimics promote infections caused by EV71 and VSV, while the application of inhibitors effectively hampers their replication (100). Overall, miR-548 seems to weaken the host’s antiviral response by directly targeting IFN-λ1, thereby facilitating the replication of EV71 and VSV, making it a potential candidate for antiviral therapies.

This modulation of IFN induction by miRNAs directly impacts downstream signaling, as discussed next.

#### 3.2.4 Targeting IFN receptors by viral-regulated miRNAs

IFNs are a class of naturally occurring proteins produced by various cell types, including fibroblasts, NK cells, leukocytes, and epithelial cells, in response to viral infections (101). The action of IFNs is initiated when they bind to specific receptors on target cells (102). These receptors are found across different tissues such as the endocrine, immune, and central nervous systems, and they are expressed on the surface of various cells, including monocytes, macrophages, T lymphocytes, glial cells, and neurons. The structure of IFN receptors is characterized by an extracellular domain that interacts with IFNs and an intracellular kinase domain activated upon dimerization induced by ligand binding (101).

The IFN-I receptor, comprising IFNAR1 and IFNAR2, is a transmembrane glycoprotein belonging to the class II family of cytokine receptors. In contrast, the IFN-III receptor system comprises two separate proteins: IL-10 receptor-β and IL-28 receptor-α. On the other hand, the type II interferon receptor is formed by the IFNγR1 and IFNγR2 subunits, which possess unique structural and functional characteristics when compared to both type I and type III receptors (103). Research conducted by Jarret et al. revealed that HCV infection leads to the aberrant expression of miR-208b and miR-499a-5p, in infected hepatocytes. These microRNAs, which originate from myosin genes, target and inhibit the production of IFNL2/3, members of the IFN-III gene family, thereby facilitating viral persistence. Furthermore, the study indicated that miR-208b and miR-499a-5p also downregulate IFN-I signaling in HCV-infected hepatocytes by reducing the expression of the IFN-I receptor chain, IFNAR1 (16). In another investigation, Zhang et al. reported that during RSV infection, levels of miR-29a increase, which subsequently targets the 3’ UTR of the IFNAR1 gene and leads to diminished expression of IFNAR1. Moreover, the RSV non-structural protein 1 (NS1) has been shown to inhibit the expression of IFNAR1 at both the RNA and protein levels in the A549 human lung adenocarcinoma cell line (104). MiR-29a functions as a negative regulator of IFNAR1 and plays a significant role in promoting RSV NS1-induced viral replication. Similarly, miR-30c contributes to porcine reproductive and respiratory syndrome virus (PRRSV) evasion of the antiviral innate response by targeting IFNAR2 (105).

#### 3.2.5 Targeting JAK/STAT components by viral-regulated miRNAs

In the canonical IFN signaling pathway, all types of IFNs activate transcriptionally active STAT1 through phosphorylation at tyrosine 701, a process facilitated by Janus kinases (JAKs) associated with the IFN receptor. Specifically, the IFNγ receptor utilizes JAK1 and JAK2 to phosphorylate STAT1, resulting in its homodimerization. The homodimerized form of STAT1, commonly known as gamma interferon-activated factor (GAF), translocates to the nucleus where it binds to gamma interferon-activated sites (GAS) in the promoters of ISGs. This binding promotes the expression of these genes, facilitating the cellular response to gamma interferon (106). In contrast, stimulation with type I or type III interferons induces the phosphorylation of both STAT1 and STAT2 through the action of TYK2 and JAK1. This leads to the formation of heterodimers that associate with IRF9, resulting in the creation of a transcriptionally active complex known as ISGF3. The ISGF3 complex binds to ISRE located in a different subset of ISGs, thereby regulating their gene expression (106–108).

Despite considerable research efforts, the detailed mechanisms governing IFN signaling pathways remain largely elusive. Recent studies have confirmed the role of miRNAs in modulating these pathways, particularly through interaction with the JAK/STAT signaling cascade. For example, Zhao et al. (109) demonstrated that miR-93 specifically targets JAK1, inhibiting the antiviral effects of IFN-I against IVA. The inhibition of miR-93 resulted in a marked reduction of IVA replication *in vivo* and contributed to improved survival rates in affected mice. Notably, miR-93 levels were found to be downregulated in patients infected with IVA, and its overexpression was shown to facilitate increased viral replication by negatively regulating the IFN-JAK-STAT pathway (109, 110). Upon recognizing an RNA virus infection, the host activates the RIG-I/JNK pathway, which correlates with decreased expression of miR-93.

Additionally, HCV infection induces the upregulation of miR-373, which negatively impacts IFN signaling by directly targeting JAK1 and IRF9. Conversely, silencing miR-373 leads to enhanced expression of IFN-stimulated genes and a reduction in HCV replication (111). HCV-mediated elevation of miR-373 also prevents the phosphorylation of STAT1, essential for the formation of the ISGF3 complex and the subsequent expression of ISGs (111). Furthermore, the overexpression of miR-30c, driven by PRRSV, further contributes to viral replication by suppressing IFN-I production through targeting of JAK1 (112).

These alterations in signaling influence the expression of IFN-stimulated genes (ISGs), the effector arm of the IFN response.

### 3.3 miRNAs modulating IFN-stimulated genes

**Figure 4:**
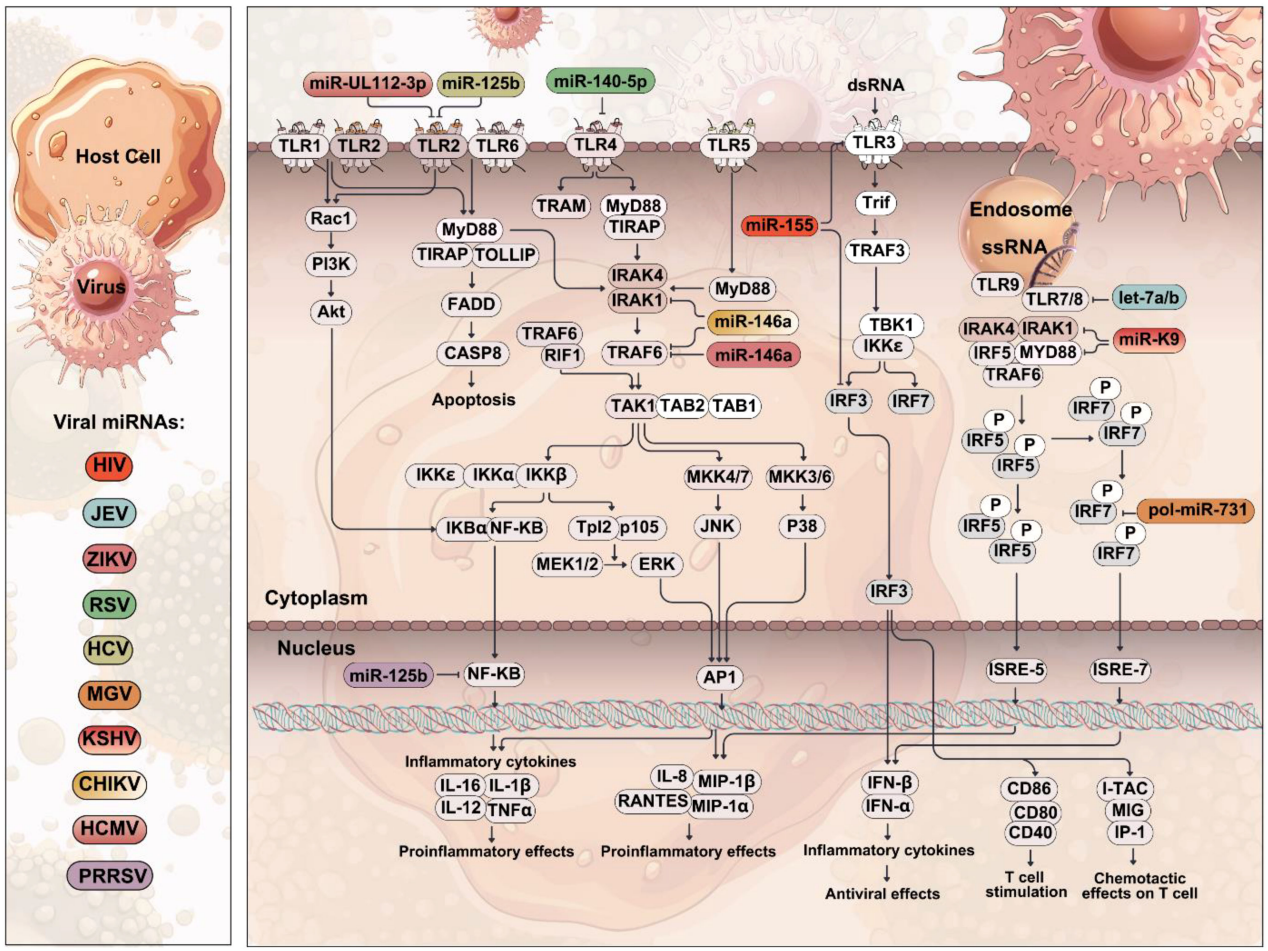
Role of TLRs in viral infection. A schematic representation of viral invasion and the activation of TLRs. Viruses bind to host cell receptors and are internalized, releasing viral genetic material in the cytoplasm. TLRs detect viral pathogen-associated molecular patterns (PAMPs), leading to the activation of downstream signaling cascades, ultimately inducing the production of pro-inflammatory cytokines and IFN-I. Viral miRNAs can target and inhibit TLR-mediated signal transduction and the subsequent induction of inflammatory cytokines, thereby preventing the development of an effective host immune response.

As previously discussed, the signal transduction that follows the interaction of IFN-I with its receptor, IFNAR, is mediated by JAKs and STAT proteins. This cascade culminates in the nuclear translocation of the transcription factor complex known as ISGF3, which is composed of IRF9, phosphorylated STAT1, and STAT2. This complex plays a critical role in inducing numerous ISGs (113). The ISGs encode a wide variety of proteins that serve multiple biological functions, which can effectively obstruct various stages of the viral life cycle, including entry, translation, replication, assembly, and dissemination. Moreover, certain ISGs possess immunomodulatory properties, contributing to the recruitment of leukocytes and the priming of the adaptive immune response. These multifaceted roles underscore the significance of ISGs in the host’s defense against viral infections. In a broader sense, an ISG is any gene whose expression is induced by the signaling cascade initiated by interferon molecules [for a recent review, see (114) and (115)]. Recent breakthroughs in RNA-sequencing (RNA-seq) technology have facilitated the discovery of ISGs across a range of cell lines by quantifying the transcriptomic response to interferon stimulation. The INTERFEROME database has been continuously updated with the findings of these gene profiling studies, providing a comprehensive repository of ISGs (116). However, the expression of ISGs exhibits a more complex reality. A subset of these genes are direct transcriptional targets of IRF3/7 and can be induced either independently or in conjunction with downstream interferon signaling pathways ***(*** **Figure 4** ***)*** (117). In addition, a distinct subset of ISGs displays both basal expression and interferon-induced upregulation, while others exhibit cell-type-specific regulation, demonstrating the complex and heterogeneous nature of ISG expression (113, 118). Furthermore, there exist three types of IFNs, with types I and III being the classical antiviral IFNs. Although type I and III IFNs interact with distinct receptors, they converge on the same signaling pathway mediated by JAK-STAT, ultimately inducing a shared repertoire of ISGs. Notably, the type I and III IFN signaling pathways are distinguished by differences in expression kinetics and cell-type specific expression patterns of their cognate receptors (114). The precise regulation of ISG expression is critical, as imbalances in the IFN-I response can culminate in the development of interferonopathies or systemic inflammation, which can have severe and far-reaching detrimental effects on the organism (119). miRNAs target specific ISGs, altering their antiviral effects.

#### 3.3.1 Myxovirus resistance

The Myxovirus resistance (Mx) genes, which are evolutionarily conserved across most vertebrates, are induced by IFN production and serve as antiviral effectors, showcasing a remarkable specificity for inhibiting the replication of RNA viruses (120). Human cells contain two distinct Mx proteins, Mx1 (commonly referred to as MxA) and Mx2 (MxB), which are members of a specialized family of dynamin-like large GTPases. These proteins are closely related to the dynamin GTPase family, sharing both a common ancestry and structural homology (113). Mx1 is recognized for its broad-spectrum antiviral activity, which occurs at an early post-entry stage in the viral life cycle, specifically before genome replication takes place. Studies have shown that Mx1 has the ability to sequester incoming viral components, including nucleocapsids, thereby thwarting their progression to designated cellular locations where they could facilitate infection. Recent advancements in unveiling the structure of Mx1 have provided valuable insights into its mechanism of action, enhancing our understanding of the molecular processes that underlie its antiviral capabilities (113, 121).

In addition to its direct antiviral properties, Mx1 expression and functionality are modulated by miRNAs, which play a significant role during viral infections. For example, miR-141 has been implicated in the negative regulation of both MxA and STAT3 expressions, thereby promoting the replication of IAV by inhibiting IFN signaling pathways (122). Similarly, elevated levels of miR-650 have been shown to facilitate IAV replication in primary human monocyte-derived dendritic cells (MDDCs) by negatively influencing the expression of several ISGs, including MxA and IFIT2. Conversely, the downregulation of miR-650 has been associated with an increase in MxA expression, potentially contributing to the establishment of an antiviral state by hindering viral replication (123). These findings underscore the intricate interplay between Mx proteins, miRNAs, and the host antiviral response, highlighting the potential for targeting these regulatory pathways in developing therapeutics against viral infections.

#### 3.3.2 Interferon-induced transmembrane proteins

The interferon-induced transmembrane (IFITM) proteins represent a key family of antiviral restriction factors that are constitutively expressed across various cell types, particularly in barrier epithelial cells, where they play a crucial role in limiting viral replication. The expression of human IFITM1, IFITM2, and IFITM3 is markedly augmented by both type I and type II interferons, emphasizing their vital contribution to the host’s antiviral defenses (124, 125). Among the IFITM proteins, IFITM1 has demonstrated significant antiviral activity by obstructing the entry of a broad spectrum of RNA viruses, including the H1N1, IAV, Dengue virus, West Nile virus, HCV, and HIV (126–128). The antiviral mechanisms attributed to IFITM proteins are thought to involve several steps, such as inhibiting viral binding to cellular receptors, blocking endocytosis, and modulating the activity of pattern recognition receptors that initiate downstream signaling pathways upon detecting viral infections (128). These proteins interfere with the fusion of viral membranes with cellular endosomal vesicular membranes by disrupting hemifusion processes. They reduce membrane fluidity and curvature, which are critical for effective membrane fusion. Additionally, these proteins may alter the balance of intracellular cholesterol, further impacting the ability of viruses to successfully enter and replicate within host cells. As a result, they play a crucial role in the cellular defense against viral infections (129, 130).

While IFITM proteins are primarily recognized for their antiviral functions, emerging evidence suggests they may also enhance viral infections under specific conditions. For example, both IFITM1 and IFITM3 can modestly promote the infection of various cell types by human papillomavirus 16 (HPV-16) (20, 131). Additionally, research by Zhao et al. has shown that interferons (IFN-I IFN-II, and IFN-III) can significantly increase the susceptibility to human coronavirus HCoV-OC43 by upregulating IFITM protein expression. They demonstrated that overexpression of IFITM3 substantially enhances the susceptibility of Huh7.5 cells to HCoV-OC43 infection (132).

The IFITM family also influences the infection process of other viruses. For instance, Hussein and Sakula recently highlighted that overexpressing IFITM1 can enhance the infection of target cells by KSHV, whereas silencing IFITM1 results in reduced infection rates. They found that IFITM1 similarly enhances infection by EBV and herpes simplex virus 2 (HSV-2). Notably, they identified that miR-36 targets the 3’ UTR of IFITM1, with overexpression of miR-36 inhibiting KSHV infection through downregulation of IFITM1. Conversely, inhibiting miR-36 allows enhanced viral infection, pointing to a post-binding regulatory role for miR-36 in viral entry. The regulation of IFITM1 by miR-36 was shown to be conserved across KSHV, EBV, and HSV-2, indicating a shared mechanism of miRNA-mediated regulation that facilitates viral internalization (133, 134).

Additionally, a significant subset of ISGs plays a role in inhibiting viral translation, with the IFN-induced protein with tetratricopeptide repeats (IFIT) family representing a notable group (113). In this regard, Buggele and Horvath (12) proposed a model regarding the regulation of host miRNA and IFIT1/ISG56 during Sendai virus (SeV) infection. Their findings indicate that SeV infection triggers an antiviral response that leads to IFN-I production, which activates the JAK-STAT-ISGF3 signaling cascade to induce the expression of ISGs, including IFIT1/ISG56 and miR-203. miR-203 can initially be induced by pre-existing proteins under the influence of IFN; however, sustaining its expression may require the ongoing synthesis of new signaling proteins, potentially involving members of the IRF family (12). Collectively, the elevated levels of miR-203, promoted by IFN-I during SeV infection, can facilitate viral replication by suppressing the antiviral effects of IFIT1/ISG56. Conversely, miR-1307 and miR-130a have been shown to restrict replication of FMDV (135) and HCV (136), respectively, by positively regulating the expression of IFN-I and several ISGs. This complex interplay underscores the multifaceted roles of ISGs and miRNAs in the host’s antiviral response, emphasizing both their protective and potentially facilitating effects on viral infections.

#### 3.3.3 The tripartite motif proteins

The tripartite motif (TRIM) protein family consists of more than 76 diverse members found in humans and mice, showcasing a wide array of functions, including the activity of E3 ubiquitin ligases (137). Recent research has demonstrated that TRIM-containing proteins play a vital role in multiple facets of the host immune response. The TRIM protein family, characterized by their function as ubiquitin E3 ligases, possesses a unique tripartite motif at their N-terminus. This motif is composed of three structural domains: a RING domain, one or two B-box domains, and a coiled-coil region (138). The RING domain equips TRIM proteins with E3 ubiquitin ligase activity, enabling the ubiquitination of target proteins. This post-translational modification affects various protein functions, including movement within the cell, stability, localization, interactions with other proteins, and enzymatic activation (139). TRIM proteins are essential in the host immune response, as they modulate the activity of important signaling molecules through ubiquitination, which boosts immunity. Moreover, they help hinder viral infections by targeting viral proteins for degradation, preventing their buildup and disrupting the viral lifecycle (137). Importantly, TRIM25 is a vital contributor to the RIG-I-mediated antiviral pathway, serving as a key component of the host’s innate immune defense against viral infections (140). Upregulation of TRIM25 has been found to significantly repress the replication of Coxsackievirus B3 (CVB3), whereas silencing TRIM25 expression leads to an increase in viral titers (141). In response to TRIM25-enhanced antiviral immunity, CVB3 has been found to evade this response by upregulating miR-30a expression. This microRNA targets and downregulates TRIM25, thereby disrupting TRIM25-mediated ubiquitination of RIG-I, which in turn impairs IFN-β activation and production. Consequently, the virus is able to enhance its replication by subverting the host’s antiviral response (141). PRRSV infection has been found to downregulate TRIM22 expression by upregulating the miR-376b-3p and miR-136. These miRNAs specifically target TRIM22, thereby negatively regulating the host’s innate antiviral responses and promoting PRRSV replication (15). Furthermore, miR-27a is a great contributor in promoting VSV replication in macrophages by negatively regulating IFN-I production. This is achieved through the targeting of TRIM27 and Siglec1, key components of the innate immune response. Notably, IFN-I induction can downregulate miR-27a expression, leading to an increase in Siglec1 and TRIM27 expression. This feedback loop subsequently inhibits IFN-I production, thereby dampening the antiviral innate response (142).

#### 3.3.4 Oligoadenylate synthetase

The oligoadenylate synthetase (OAS) family, comprising DNA sensor cGAS and RNA-sensing OAS/OASL proteins, is a critical class of ISGs that plays a key role in the host’s antiviral defense through the recognition of viral RNA and DNA (143). OAS proteins detect cytosolic dsRNA, triggering RNase L activation to halt viral replication and establish an antiviral state. The importance of this pathway is evident from the diverse strategies viruses have developed to evade OAS-mediated antiviral responses (143). Zhu et al. (144)., found that SARS-CoV-2-encoded miR-ORF1ab-2-5p targets and regulates OAS1 and OAS2, antiviral enzymes induced by interferon. The stable binding of miR-ORF1ab-2-5p to the pathogenic risk allele rs7967461(C) of OAS1 leads to a decrease in OAS1 expression. This reduction facilitates SARS-CoV-2 replication by impairing the IFN-I signaling pathway (144). In a related context, Bouvet et al. discovered that the EBV-encoded miR-BART1 represses the expression of OAS2, thereby facilitating the development of viral infection (145). Zhao et al. demonstrated that the suppression or knockdown of endogenous miR-340 prevents IAV infection, whereas the enforced expression of miR-340 significantly enhances virus replication (146). Also, they discovered that miR-340 impairs cellular antiviral immunity by targeting OAS2, a crucial component of the antiviral response (143). Taken together, these findings suggest that host cells may counterbalance viral loads by modulating miRNA pathways, which may offer novel therapeutic opportunities for the treatment of viral infections.

#### 3.3.5 ISG15

ISG15 is a paradigmatic example of an ISG, exhibiting strong induction in response to interferon stimulation. As previously indicated, ISG15 is a ubiquitin-like protein capable of forming covalent bonds with target proteins through a process known as ISGylation (113). ISG15 appears to have a complex role in regulating various cellular responses, which contributes to the establishment of an antiviral state (147). It restricts viral replication by disrupting the translation and exocytosis machinery that viruses exploit for their propagation. Additionally, it impedes the budding of virus-like particles by inhibiting essential enzymes involved in this process (147). Most viral proteins require oligomerization or complex formation to function effectively. ISGylation of these viral proteins acts as an effective antiviral strategy, as the attachment of the ISG15 protein introduces steric hindrance. This steric hindrance prevents further oligomerization of the ISGylated proteins when they are incorporated into complexes. Consequently, the ISGylation of even a limited number of viral proteins can lead to a substantial inhibitory effect on viral replication and activity (147). Beyond its role in intracellular immune responses, ISG15 is critical for extracellular immune responses, highlighting its multifaceted participation in the immune system (147).

Viruses have devised various strategies to evade the antiviral action of ISG15, one of which includes the alteration of host microRNA levels to specifically target ISG15 itself. A notable example is the upregulation of miR-130a, which boosts the expression of IFN-I and ISG15, thereby triggering an innate immune response that restricts HCV replication by restoring a functional innate immune state (136). Conversely, HCV infection leads to a significant decrease in miR-130a expression in infected cell lines, suggesting that the virus may exploit this downregulation to evade the host’s innate immune response (148). Ectopic miR-130a overexpression suppresses HCV replication by stimulating IFN-I (IFN-α/β) and ISG15 expression, thereby promoting an innate immune response that restricts viral replication (136).

#### 3.3.6 Interferon regulatory factor

The interferon regulatory factor (IRF) family plays an essential role in regulating type I interferons (IFN-α/β) and ISGs by directly interacting with and binding to the ISRE sequence found in the promoter regions of these genes. Originally recognized as a vital regulator of the IFN-α/β promoter, the binding of IRF to ISRE is important for the transcriptional activation of these genes (149). The IRF family includes three primary members IRF3, IRF5, and IRF7 which are vital for producing IFN-I following the activation of pathogen recognition receptors that recognize viral RNA and DNA. In contrast, IRF9 is critical for regulation of the expression of genes induced by interferons. Additionally, IRF4, IRF8, and IRF5 play vital parts in the development and differentiation of myeloid cells, thus impacting inflammatory responses (150). The regulation of IRF levels and activity is crucial, as aberrations in either can lead to imbalanced immune responses and potentially trigger autoimmune diseases. Interestingly, studies have demonstrated that IRF regulation by miRNAs during viral infections can facilitate viral replication by suppressing the IFN pathway. Notably, the upregulation of miR-373 during HCV infection has been shown to impair the IFN antiviral response by targeting IRF5, thereby enabling viral replication (151) and IRF9 (111). Similarly, Zhang et al. (152) discovered that miR-731 disrupts the IFN-I response induced by megalocytiviruses and suppresses IFN expression at both the mRNA and protein levels. Their findings indicate that miR-731 modulates the IFN-I response by targeting IRF7. Furthermore, they observed that ectopic expression of miR-731 significantly downregulates the expression of IFITIM1, ISG15, Mx, and viperin. Conversely, silencing miR-731 leads to increased expression levels of ISG proteins (152).

The Megalocytivirus-induced pol-miR-731 plays a crucial role in facilitating viral replication by suppressing ISG expression levels through the targeted degradation of IRF7. Furthermore, miR-155 has been shown to contribute to HIV’s evasion of the host’s innate immune response by targeting IRF3. In contrast, miR-722 has been found to restrict CyHV-3 replication by positively regulating IFN expression through the targeted degradation of viral ORF89, which acts as an inhibitor of IRF3 (153). MiR-34a has been identified as an antiviral microRNA that potentiates the IFN-I response by positively modulating the expression level of IRF3 through the targeting of Wnt signaling during Flavivirus infection (154). Notably, IRF-3 has been found to interact with the co-activator proteins CREB binding protein (CBP) or p300 (CBP/p300), a mechanism that plays a crucial role in modulating IFN production. Specifically, a compactly folded 46-residue domain within CBP, known as the IRF3 binding domain (IBiD), is responsible for binding to the C-terminal region of IRF-3 (155). Upon activation, IRF3 forms a complex with the co-activator proteins CBP/p300, leading to the initiation of transcription of the IFN-β gene (156, 157). Phosphorylation at serine residues 386 and 396 on IRF3 is crucial for its activation and subsequent interaction with the co-activator CBP (158, 159). Following phosphorylation and dimerization, activated IRF3 undergoes nuclear translocation, where it interacts with and recruits the co-activators CBP and/or p300, thereby achieving full activation (156, 157, 160). Subsequently, the IRF3-CBP/p300 complex binds to the PRD I/III elements within the IFN-β promoter, thereby triggering transcriptional activation and inducing the production of IFN-I-β (161).

Studies have demonstrated that the EBV-encoded BART16 protein suppresses the antiviral IFN response to latent EBV infection by targeting the CBP (162). The EBV-encoded miR-BART16 directly targets CBP, leading to its downregulation in EBV-transformed B cells and gastric carcinoma cells. Notably, miR-BART16 has been shown to abrogate the production of ISGs in response to IFN-α stimulation by inhibiting CBP activity. Furthermore, miR-BART16 has been found to suppress the anti-proliferative effect of IFN-α on latently infected Burkitt lymphoma (BL) cells. By blocking the IFN-I -induced antiviral response, miR-BART16 creates a mechanism for facilitating the establishment of latent EBV infection and enhancing viral replication (162).

Transcriptome analyses have indicated that IRF1 is responsible for regulating a substantial number of genes, including approximately 300 antiviral ISGs such as OAS2, BST2, and RNASEL. The inhibition of IRF1 has been associated with increased susceptibility of cells to various viruses, including HCV and herpes simplex virus-1. Furthermore, previous research has shown that type I interferons are downregulated by miRNAs that target IRF1 during viral infections. For instance, Zhang et al. demonstrated that IRF1 is a direct target of miR-132-3p during H1N1 IAV infection. Significantly, the antiviral effect of miR-132-3p knockdown on IAV replication was eliminated when IRF1 was inhibited, indicating that the miR-132-3p inhibitor reduces IAV replication by directly targeting IRF1 and elevating the antiviral response (163). Similarly, overexpression of miR-302 induced by PRRSV negatively regulates IRF1, leading to a reduction in IFN-β production and facilitating PRRSV replication (164). The inability of the host to effectively generate type I interferons in response to JEV infection is associated with a heightened risk of lethal disease (165). Hazra and colleagues (166) demonstrated that JEV-induced expression of miR-301a inhibits IFN-I production by reducing IRF1 levels. In mouse neurons, the neutralization of miR-301a was found to restore the innate immune response by promoting IFN-β production, thereby limiting viral spread. Inhibition of miR-301a in the mouse brain resulted in the restoration of IRF1 levels, increased IFN-β generation, and a reduction in JEV replication, ultimately improving survival outcomes for the mice (166). Additionally, miR-373 and miR-23a have been shown to enhance HSV-1 replication by specifically suppressing IRF1, a key transcription factor necessary for the induction of antiviral genes.

### 3.4 miRNAs targeting negative regulators of the IFN pathway

Negative regulators such as SOCS, CUEDC2, USP18, USP15, and the ATG5-ATG12 complex suppress IFN signaling to prevent excessive immune activation. Notably, miRNAs can counteract this suppression by directly targeting these regulators, thereby enhancing antiviral responses—a mechanism explored in detail below.

The JAK/STAT signaling pathways, which are crucial for mounting antiviral immune responses and maintaining immune balance, are strongly regulated by negative regulators, including SOCS and CUEDC2 to prevent excessive or prolonged activation and ensure optimal viral clearance (167, 168). The suppressor of cytokine signaling proteins (SOCS) protein family consists of a range of intracellular proteins, including CIS, SOCS1/2/3/4/5/6/7. Upon activation, STATs undergo a process of homodimerization, followed by translocation to the nucleus, where they induce the transcriptional activation of SOCS genes. The resulting SOCS proteins subsequently interact with phosphorylated JAK and its receptor, thereby modulating the JAK/STAT signaling pathway through three distinct mechanisms (169). It has been observed that certain miRNAs can overcome the inhibitory effect of SOCS proteins on IFN production, thereby leading to an increase in IFN production and subsequently inhibiting viral infection (**Supplementary Table 1**). MiR-30a-5p has been found to suppress transmissible gastroenteritis coronavirus (TGEV) infection by augmenting the antiviral signaling pathway triggered by IFN production. This is achieved by directly targeting and inhibiting the expression of SOCS1 and SOCS3, two key negative regulators of the IFN signaling pathway (170). TGEV has been shown to counteract the antiviral effects of miR-30a-5p by suppressing its expression. As a result, the negative regulators of the JAK-STAT signaling pathway, SOCS1 and SOCS3, are upregulated, leading to a blunted antiviral response (170).

Recently, Yan et al. (171) demonstrated that miR-221 directly targets SOCS1 and suppresses its expression at both the mRNA and protein levels. Notably, the overexpression of miR-221 has been shown to both inhibit replication of HCMV and enhance the production of IFN-I and ISGs. In contrast, the reintroduction of SOCS1 mitigates the effects of miR-221 on HCMV replication, suggesting a role for SOCS1 in counteracting the antiviral effects mediated by miR-221 (171). Furthermore, miR-221 has been observed to facilitate the phosphorylation and activation of NF-κB by inhibiting the expression of SOCS1. This implies that miR-221 may modulate the NF-κB signaling pathway, which is an essential transcription factor involved in various cellular functions, including inflammation and immune responses (171). Similarly, miR-155 has demonstrated antiviral effects against VSV in macrophages by promoting the production of IFN-I through the targeting of SOCS1. This data indicates that miR-155 might play a critical role in the host’s defense mechanisms against VSV infection (172). Gao et al. (173) further reported that reductions in SOCS3 levels positively influence the antiviral efficacy of endogenous IFN-I against hepatitis B virus (HBV). Furthermore, our research indicates that the downregulation of miR-122 during HBV infection results in the reduced expression of interferons. This reduction promotes HBV replication and may play a role in viral persistence and the development of hepatocellular carcinoma (173). miR-221 and miR-30c-5p exert a positive regulatory effect on the IFN-I pathway by targeting SOCS1, subsequently suppressing the replication of HPV-16 (174) and PEDV (175), respectively. Zhai et al. (176) demonstrated that the borna disease virus (BDV) protein directly suppresses miR-155 expression in infected cells, leading to increased inhibition of IFN-Is in these cells. Notably, when miR-155 is overexpressed, this inhibition is further augmented. Additionally, miR-155 is found to promote IFN-I production by targeting SOCS1 and SOCS3, thereby restricting BDV infection. Consequently, the persistent infection with BDV leads to the suppression of IFN-Is through the downregulation of miR-155, underscoring the crucial role of miR-155 in immune regulation during BDV persistent infection (176). CUEDC2, a negative regulator of the JAK1-STAT3 pathway, has been found to be targeted by miR-324-5p at its 3’ UTR. The study shows that miR-324-5p enhances the host’s antiviral response by inhibiting CUEDC2, thereby limiting H5N1 replication. This highlights the significance of miR-324-5p as a critical contributor in modulating the host’s immune response to H5N1 infection (177). Ubiquitin Specific Peptidase (USP) 18 (178) and 15 (179) are additional negative regulators of the type 1 IFN pathway, which can be modulated by miRNAs. In this context, the ectopic expression of miR-130a has been shown to suppress HCV replication by positively regulating IFN-I-α/β production through the negative regulation of USP18 expression levels (136). Notably, miR-26a has been found to enhance IFN-I production and restrict the replication of various viruses, including HEV, VSV, and SeV, by targeting USP15 (180).

The ATG5-ATG12 complex, an essential element of the autophagy machinery, has been reported to negatively regulate IFN-I production in MEFs, thereby facilitating VSV replication (181). Additionally, the importance of ATG5 in IFN-I production has been highlighted in plasmacytoid dendritic cells (pDCs) infected with VSV, underscoring its role in antiviral responses (182). Besides, Duan et al. (183) found that ATG5 promotes HCV replication by downregulating the expression of ISGs. In an interesting counteraction, the overexpression of miR-130a was shown to decrease ATG5 levels, which in turn disrupts the conjugation of the ATG5-ATG12 protein complex. This disruption ultimately leads to the inhibition of HCV infection through the substantial upregulation of several key ISGs, including MX1 and OAS3 (183). These findings suggest a complex interplay between autophagy-related proteins, miRNAs, and the antiviral immune response, highlighting the potential for targeted therapeutic strategies in managing viral infections.

This tight regulation by miRNAs underscores their double-edged role in viral infections: while some miRNAs facilitate immune evasion by suppressing IFN pathways, others reinforce antiviral defenses by enhancing ISG expression—a dichotomy that highlights their context-dependent impact on infection outcomes.

## 4 Harnessing miRNAs: emerging therapeutic strategies for antiviral immunity

Recent studies have underscored the promising potential of miRNAs in combating viral infections by enhancing the body’s antiviral immune response. Therefore, this section delves into the current applications of miRNAs as therapeutic targets, highlighting three primary strategies: miRNA antagonists (antagomiRs), miRNA mimics, and small-molecule miRNA modulators. These approaches hold significant promise for advancing antiviral treatment paradigms.

AntagomiRs are chemically modified oligonucleotides designed to block specific miRNAs that help viruses replicate or weaken the immune response (184). For example, miravirsen targets miR-122, which is essential for hepatitis C virus (HCV) stability, and has shown promise in clinical studies by forming a stable complex that prevents the virus from thriving (185). Another example is miR-146a antagomirs, which have been used in mouse models to reduce influenza A virus (IAV) infection and mitigate severe immune reactions (186), like cytokine storms seen in diseases such as COVID-19. These antagomiRs are effective at low doses and.

### 4.1 miRNA antagonists (AntagomiRs): detailed mechanisms and applications

AntagomiRs are chemically modified antisense oligonucleotides designed to specifically inhibit the function of target miRNAs (184). Their role in antiviral therapy by targeting miRNAs that facilitate viral replication or suppress host immunity has been highlighted. For instance, miR-122 is noted as a critical miRNA for HCV replication, stabilizing the viral genome. Miravirsen, a locked nucleic acid (LNA)-modified DNA phosphorothioate antisense oligonucleotide, is cited as an example that binds to mature miR-122, forming a stable heteroduplex that impedes its function, thereby inhibiting HCV infection (185, 187). This approach has been explored in clinical settings, demonstrating efficacy in reducing viral load.

### 4.2 miRNA mimics: enhancing host antiviral defenses

MiRNA mimics are synthetic RNA molecules designed to replicate the function of endogenous miRNAs that suppress viral replication, thereby enhancing the host’s antiviral defense mechanisms (188–190). One strategy involves utilizing miRNA mimics to boost the levels of natural miRNAs that naturally inhibit viral activity. For example, Ivacik et al. (191), demonstrated that lentiviral vectors carrying miRNAs derived from pri-miR-31, under the control of a liver-specific promoter, efficiently suppress HBV replication by targeting host pathways that limit viral spread (191). This approach leverages gene delivery systems to ensure effective expression of the mimic at the site of infection (184).

Additionally, HBV-miR-3, an HBV-specific miRNA, inhibits HBV replication by downregulating SOCS5 and activating the JAK/STAT signaling pathway and interferon-induced antiviral effects (192). This mechanism enhances the host’s innate immune response, offering a targeted strategy to combat HBV. Therefore, miRNA mimics provide a precise way to modulate host-virus interactions, reinforcing natural antiviral capabilities. However, challenges such as delivery efficiency and potential off-target effects are acknowledged, requiring innovative solutions like viral vectors, liposomes, and nanoparticles to ensure mimics reach infected cells without triggering adverse immune responses (184).

### 4.3 Small-molecule miRNA modulators: emerging but understudied

Small-molecule miRNA modulators represent an innovative and promising strategy in the realm of miRNA-based therapeutics, particularly for antiviral immunity. These compounds are designed to either enhance or inhibit the expression of specific miRNAs by interacting with their biogenesis pathways or directly modulating their activity (193). This approach leverages the unique ability of small molecules to cross cell membranes via free diffusion, making them more accessible to intracellular targets compared to traditional nucleic acid-based therapies (194).

Small-molecule modulators function through various mechanisms to influence miRNA pathways. One primary method involves transcriptional activation, where these molecules can upregulate the expression of tumor-suppressive miRNAs that have been downregulated in disease states. Conversely, small-molecule inhibitors can suppress the activity of overexpressed oncogenic miRNAs. By blocking the pathways responsible for miRNA maturation or binding directly to mature miRNAs, these inhibitors can prevent the miRNAs from exerting their effects on target mRNAs (193–195). This dual capability—activation and inhibition—makes small molecules highly versatile tools in therapeutic development.

The advantages of using small-molecule modulators include their stability, ease of synthesis, and potential for oral administration, which collectively contribute to better patient compliance and reduced treatment costs (193). Additionally, their ability to permeate cell membranes without the need for complex delivery systems circumvents many of the challenges associated with nucleic acid-based therapies, such as poor cell-permeability and susceptibility to degradation (194). However, several challenges remain. The multifaceted roles of miRNAs in cellular processes mean that modulation can lead to unintended off-target effects, affecting multiple pathways and potentially causing adverse reactions (194). Furthermore, ensuring the specificity and selectivity of small molecules is crucial to avoid disrupting non-targeted miRNAs, which could exacerbate disease conditions or trigger new pathologies (196–198).

Despite these challenges, the exploration of small-molecule miRNA modulators has opened new avenues for treating viral infections, including those caused by emerging viruses like SARS-CoV-2 (194). Understanding the precise roles of specific miRNAs in viral pathogenesis is essential for developing targeted therapies that can inhibit viral replication and mitigate disease severity (193, 194).

Future research should focus on refining the specificity of these modulators and exploring combination therapies that integrate small molecules with other therapeutic modalities, such as miRNA mimics or antagomirs (194). Advances in computational modeling and high-throughput screening technologies will further facilitate the discovery of novel small-molecule modulators with enhanced efficacy and safety profiles (193).

### 4.4 Challenges and future directions

Despite these promising therapeutic potentials, challenges remain. The inherent instability of RNA molecules and the need for targeted delivery systems complicate the direct translatability of these approaches, especially in the context of pandemics like COVID-19 (194). Additionally, off-target effects and immunogenicity must be carefully evaluated to ensure safety and efficacy (199). Nevertheless, ongoing advancements in delivery mechanisms, such as hydrogel-based systems and nanocell technologies, hold promise for overcoming these hurdles (200). Delivery technologies such as viral vectors, liposomes, and nanoparticles are suggested as potential solutions, but their development requires further optimization to minimize off-target effects and immune responses (184).

Another challenge is inter-individual variability in miRNA expression, which may affect therapeutic efficacy, necessitating personalized approaches. The study also notes the risk of off-target effects, where miRNA therapies could disrupt normal cellular functions, highlighting the need for specificity in targeting (184, 201).

Overall, miRNAs offer novel and emerging targets for therapeutic intervention in viral infections. Current strategies employing miRNA antagonists, mimics, and small-molecule modulators illustrate the versatility and potential of miRNA-based therapies (199). Continued research is essential to fully harness the therapeutic capabilities of miRNAs and translate these findings into effective clinical applications.

## 5 Conclusion

During the past decade, considerable progress has been achieved in understanding the complex network through which miRNAs regulate the development and functioning of the immune system. Innovative approaches to deliver miRNA mimics and antagonists *in vivo*, particularly through viral vectors and nanoparticles, have opened new avenues for therapeutic interventions aimed at modulating pathological hematopoiesis (21, 202, 203). Moreover, techniques for genetically modifying miRNAs using transcription activator-like effector nucleases (TALENs) (204, 205) and CRISPR–Cas9-mediated cleavage (206, 207) in mammalian cells hold great promise for both elucidating fundamental miRNA mechanisms and developing novel therapeutics (208, 209).

Given the critical role of the interferon (IFN) pathway in combating viral infections, alongside miRNAs’ significant influence on the activation or repression of this pathway, we explored how miRNAs can modulate viral replication by targeting the IFN pathway (210). Selecting appropriate miRNAs for modulation—either through enhancement or inhibition—could represent a novel therapeutic strategy for addressing viral infections. Achieving effective miRNA-based interventions will necessitate a comprehensive understanding of miRNA action mechanisms in regulating the IFN pathway and the broader innate immune response.

Furthermore, the potential of miRNA-based drug delivery, especially in conjunction with IFN therapies, could provide additional tools for controlling viral infections. However, despite the promising prospects of miRNA therapeutics, several practical challenges remain. These include determining optimal administration routes, ensuring stability within the body, targeting specific tissues and cell types, and achieving the desired intracellular effects (211). As a result, only a limited number of miRNA-based drugs have progressed to clinical testing. Additionally, a thorough risk assessment of miRNA therapeutics is essential prior to *in vivo* applications to mitigate off-target effects and avoid potential miRNA overdosing. Ultimately, addressing these challenges will be crucial for translating miRNA-based approaches into effective clinical therapies.
