# Supplementary material for: Interferon and immunity: the role of microRNA in viral evasion strategies
Source: Front Immunol. 2025 May 9;16:1567459. doi: 10.3389/fimmu.2025.1567459 (PMC12101089; doi:10.3389/fimmu.2025.1567459)
Supplement: Supplementary file 1 [file Table1.docx]

**Supplementary Table 1. Role of microRNAs in the regulation of interferon in viral diseases**

| **Viral infection** | **MicroRNA (Up/Down)** | **Target** | **Inhibition/ Induction of interferon pathway** | **Note** | **Ref** |
| --- | --- | --- | --- | --- | --- |
| ***Orthomyxoviridae*** | | | | | |
| Influenza A virus (IAV) | miR-302c | NIK | Inhibition | miR-302 inhibits IFNβ expression by targeting NIK and in return, IVA increases IFNβ levels by negatively regulating miR-302c. | ^1^ |
| IAV (H1N1) | miR-194 | FGF2 | Inhibition | miR-194 contributes to IAV replication by inhibiting IFN production and RIG-I signaling through targeting FGF2. | ^2^ |
| IAV | let-7 (Down) | RPS16 | Induction | Let-7 inhibits IAV replication by promoting interferon type 1 production by targeting RPS16. | ^3^ |
| IAV | miR-221 | SOCS1 | Induction | miR-221 inhibits IAV replication by promoting IFN‑α and IFN‑β production through targeting SOCS1. | ^4^ |
| IAV | miR-26a | USP3 | Induction | miR-26a alleviates IAV replication by activating type I IFN signaling pathway through targeting USP3. | ^5^ |
| IAV | miR-449b | HDAC1 | Induction | miR-449b promotes antiviral responses by positively regulating IFN-β expression through targeting HDAC1. | ^6^ |
| Influenza | miR-30 | SOCS1, SOCS3, and NEDD4 | Induction | miR-30 alleviates influenza replication by inducing antiviral immune response by targeting SOCS1, SOCS3, and NEDD4. | ^7^ |
| IAV (H1N1) | miR-155-5p | - | Induction | miR-155-5p promotes antiviral immune response by increasing IFN-β production through positively regulating STAT1. | ^8^ |
| H5N6 | miR-200c | CNTN1 and USP25 | Induction | H5N6-overexpressed miR-200c restricts viral replication by promoting type I IFN production through targeting CNTN1 and USP25. | ^9^ |
| IVA | miR-483-3p | RNF5 | Induction | miR-483-3p enhances viral anti-viral response by promoting the production of type I IFN and proinflammatory cytokine through targeting RNF5. | ^10^ |
| ***Rhabdoviridae*** | | | | | |
| VSV | miRNA-548 | IFN-λ1 | Inhibition | miRNA-548 facilitates VSV replication by weakening the antiviral immune response, achieving this through the specific targeting of IFN-λ1. | ^11^ |
| VSV | miR-146a | TRAF6, IRAK1, and IRAK2 | Inhibition | VSV-induced miR-146a promotes viral replication by negatively regulating RIG-I signaling and type I IFN production through targeting TRAF6, IRAK1, and IRAK2. | ^12^ |
| VSV | miR-146a | TRAF6, IRAK1, and IRAK2 | Inhibition | VSV-induced miR-146a promotes viral replication by negatively regulating RIG-I signaling and type I IFN production through targeting TRAF6, IRAK1, and IRAK2. | ^12^ |
| VSV | miR-548 | IFN-λ1 | Inhibition | miR-548 contributes to VSV replication by suppressing the type II IFN response, specifically by targeting IFN-λ1. | ^11^ |
| VSV | miR-223 (Up) | FOXO3 | Induction | Upregulated miR-223 in VSV-infected macrophages promotes type I IFN production by targeting FOXO3. | ^13^ |
| VSV | miR-526a | CYLD | Induction | miR-526a alleviates VSV replication by positively regulating type I IFN pathway through targeting CYLD. | ^14^ |
| Rhabdovirus | miR-210 | STING | Induction | Rhabdovirus-overexpressed miR-210 enhances viral replication by negatively regulating type I IFN and inflammatory cytokines through targeting STING. | ^15^ |
| ***Herpesviridae*** | | | | | |
| HSV-1 | miR-23a | IRF1 | Inhibition | miR-23a promotes HSV-1 replication by negatively regulating IFN response through targeting IRF1. | ^16^ |
| HSV-1 | miR-373 | IRF1 | Induction | miR-373 increases HSV-1 replication by inhibiting type I IFN response through negatively regulating IRF1 level. | ^17^ |
| PRV viruses | miR-155-5p | - | Induction | The antiviral immune response to PRV is amplified by miR-155-5p through STAT1-mediated induction of IFN-β synthesis. | ^8^ |
| ***Flaviviridae*** | | | | | |
| HCV | miR-21 | IRAK1 and MyD88 | Inhibition | HCV-overexpressed miR-21 facilitates virus replication by inhibiting type I IFN prosuction through targeting IRAK1 and MAPK. | ^18^ |
| Chronic HCV | miR-155  (Down) | Tim-3 | Inhibition | Chronic HCV inhibits IFN-γ production in NK by positively regulating Tim-3 level through decreasing miR-155 expression level. | ^19^ |
| HCV | miR-182 | FOXO3 | Induction | miR-182 enhances the anti-HCV response by inducing type I IFN through targeting FOXO3 and positively regulating IRF7. | ^20^ |
| HCV | miR-130a | - | Induction | Ectopic expression of miR-130a suppresses HCV replication by positively regulating type I IFN (IFN-α/IFN -β), MxA, USP18, and ISG15. | ^21^ |
| JEV | miR-374b-5p | PTEN | Inhibition | Upregulated miR-374b-5p alleviates type I IFN response by targeting PTEN and regulating PI3K/AKT pathway during JEV infection in microglial cells. | ^22^ |
| JEV | miR-15b | RNF125 | Induction | JEV promotes type I IFN and proinflammatory cytokines by positively regulating RIG I through targeting RNF125. | ^23^ |
| JEV | miR-155 | TBK-1 | Induction | Upregulated miR-155 during JEV promotes neuroinflammation by inducing the production of IFN-β and proinflammatory cytokine through targeting TBK-1. | ^24^ |
| Dengue virus | miR-30e* | IκBα | Induction | miR-30e* alleviates dengue virus replication by enhancing NF-κB-dependent IFN production through targeting IκBα. | ^25^ |
| Dengue virus | miR-155 (Down) | Bach1 | Induction | Ectopic expression of miR-155 leads to inhibiting dengue virus replication by inducing heme oxygenase-1-mediated IFN responses through targeting bach1. | ^26^ |
| ***Hepadnaviridae*** | | | | | |
| Chronic HBV | miR-548j | ZBTB11 | Inhibition | HBV-induced miR-548j enhances viral replication by negatively regulating type I IFN through targeting ZBTB11. | ^27^ |
| ***Arteriviridae*** | | | | | |
| PRRSV | miR-3 | IRAK1 | Inhibition | PRRSV-overexpressed miR-3 facilitates viral replication by negatively regulating IFN-α production through targeting IRAK1. | ^28^ |
| PRRSV | miR-382-5p | - | Inhibition | miR-382-5p promotes PRRSV replication by negatively regulating type I IFN production. | ^29^ |
| PRRSV | miR-23 | - | Induction | miR-23 promotes antiviral immune response against PRRSV by enhancing the expression of type I IFN through activating IRF3/IRF7. | ^30^ |
| PRRSV | miR-26a | - | Induction | miR-26a enhances innate anti-viral responses by activating type I IFN. | ^31^ |
| ***Picornaviridae*** | | | | | |
| EV71 | miR-548 | IFN-λ1 | Inhibition | miR-548 facilitates EV71 and VSV replication by inhibiting type II IFN response through targeting IFN-λ1. | ^11^ |
| EV71 | miRNA-548 | IFN-λ1 | Inhibition | miRNA-548 promotes EV71 and VSV replication by alleviates antiviral immune response through targeting IFN-λ1. | ^11^ |
| FMDV | miR-4331-5p | - | Inhibition | FMDV-induced miR-4331-5p promotes viral replication by negatively regulating type I IFN pathways. | ^32^ |
| CVB3 | miR-30a | TRIM25 | Inhibition | miR-30a contributes to CVB3 replication by suppressing type I IFN production through targeting TRIM25. | ^33^ |
| ***Coronaviridae*** | | | | | |
| PEDV | miR-615 | IRAK1 | Inhibition | miR-615 contributes to PEDV replication by inhibiting type III IFN production through targeting IRAK1. | ^34^ |
| PEDV | miR-221-5p | - | Induction | miR-221-5p restricts PEDV replication by inducing type I IFN production through activating NF-κB Signaling. | ^35^ |
| ***Birnaviridae*** | | | | | |
| IBDV | gga-miR-9* | IRF2 | Inhibition | gga-miR-9* facilitates IBDV replication by inhibiting type I IFN through targeting IRF2. | ^36^ |
| IBDV | gga-miR-130b | SOCS5 | Induction | gga-miR-130b restricts IBDV replication by promoting IFN-β expression through targeting SOCS5. | ^37^ |
| ***Alloherpesviridae*** | | | | | |
| CyHV-3 | miR-155 | AMPK | Induction | miR-155 inhibits CyHV-3 replication by positively regulating IFN expression through targeting AMPK. | ^38^ |
| **Viral-encoded miRNAs** | | | | | |
| ***Herpesviridae*** | | | | | |
| EBV | BART7-3p | IFNL3 | Inhibition | EBV-encoded miR-BART7-3p contributes viral replication by inhibiting antiviral IFN-response through targeting IFNL3. | ^39^ |
| EBV | miR-BART-18 | - | Inhibition | EBV-encoded miR-BART-18 alleviates IFN signaling and maybe contributes to IFN therapy resistance in EBV-associated cancers. | ^40^ |
| KSHV | miR-K6-5 and –K9* | - | Inhibition | KSHV encoded miRNAs such as miR-K6-5 and –K9* can inhibit antiviral activity of IFN by suppressing STAT3 activation. | ^41^ |
| HSV-1 | miR-H28 | - | Induction | Exosomal HSV-1 miR-H28 restricts HSV-1 spread from cell to cell by inducing IFN-γ. | ^42^ |
| HSV-1 | miR-H2-3p | DDX41 | Induction | HSV-1-encoded miR-H2-3p facilitates viral proliferation by inhibiting IFN-β production through targeting DDX41. | ^43^ |
| ***Retroviridae*** | | | | | |
| HIV | vmiR88 and vmiR99 | - | Induction | HIV-encoded vmiR88 and vmiR99 promotes TNFα release from macrophage by positively regulating TLR8. | ^44^ |
| ***Hepeviridae*** | | | | | |
| HEV | miR-A6 (miR-A6M1) | SIRP-α | Inhibition | HEV- miR-A6 (miR-A6M1) contributes to viral replication by inhibiting antiviral IFN-response by targeting SIRP-α. | ^45^ |

1. Gui, S. *et al.* Mir-302c mediates influenza A virus-induced IFNβ expression by targeting NF-κB inducing kinase. *FEBS Letters* **589**, 4112-4118 (2015).

2. Wang, K. *et al.* miR-194 Inhibits Innate Antiviral Immunity by Targeting FGF2 in Influenza H1N1 Virus Infection. *Frontiers in Microbiology* **8** (2017).

3. Wu, W., Wang, C., Xia, C., Liu, S. & Mei, Q. MicroRNA let-7 Suppresses Influenza A Virus Infection by Targeting RPS16 and Enhancing Type I Interferon Response. *Frontiers in Cellular and Infection Microbiology* **12** (2022).

4. Zhang, N. *et al.* Downregulation of microRNA‑221 facilitates H1N1 influenza A virus replication through suppression of type‑IFN response by targeting the SOCS1/NF‑κB pathway. *Mol Med Rep* **24**, 497 (2021).

5. Gao, S., Li, J., Song, L., Wu, J. & Huang, W. Influenza A virus-induced downregulation of miR-26a contributes to reduced IFNα/β production. *Virologica Sinica* **32**, 261-270 (2017).

6. Buggele, W.A., Krause, K.E. & Horvath, C.M. Small RNA Profiling of Influenza A Virus-Infected Cells Identifies miR-449b as a Regulator of Histone Deacetylase 1 and Interferon Beta. *PLOS ONE* **8**, e76560 (2013).

7. Lin, X., Yu, S., Ren, P., Sun, X. & Jin, M. Human microRNA‐30 inhibits influenza virus infection by suppressing the expression of SOCS1, SOCS3, and NEDD4. *Cellular microbiology* **22**, e13150 (2020).

8. Rai Kul, R. *et al.* MIR155HG Plays a Bivalent Role in Regulating Innate Antiviral Immunity by Encoding Long Noncoding RNA-155 and microRNA-155-5p. *mBio* **13**, e02510-02522 (2022).

9. Xu, S. *et al.* MicroRNA-200c-targeted contactin 1 facilitates the replication of influenza A virus by accelerating the degradation of MAVS. *PLOS Pathogens* **18**, e1010299 (2022).

10. Maemura, T. *et al.* Lung-Derived Exosomal miR-483-3p Regulates the Innate Immune Response to Influenza Virus Infection. *J Infect Dis* **217**, 1372-1382 (2018).

11. Li, Y. *et al.* MicroRNA-548 down-regulates host antiviral response via direct targeting of IFN-λ1. *Protein & Cell* **4**, 130-141 (2013).

12. Hou, J. *et al.* MicroRNA-146a Feedback Inhibits RIG-I-Dependent Type I IFN Production in Macrophages by Targeting TRAF6, IRAK1, and IRAK21. *The Journal of Immunology* **183**, 2150-2158 (2009).

13. Chen, L. *et al.* MicroRNA-223 promotes type I interferon production in antiviral innate immunity by targeting forkhead box protein O3 (FOXO3). *Journal of Biological Chemistry* **291**, 14706-14716 (2016).

14. Xu, C. *et al.* Downregulation of MicroRNA miR-526a by Enterovirus Inhibits RIG-I-Dependent Innate Immune Response. *Journal of Virology* **88**, 11356-11368 (2014).

15. Xu, T., Chu, Q. & Cui, J. Rhabdovirus-Inducible MicroRNA-210 Modulates Antiviral Innate Immune Response via Targeting STING/MITA in Fish. *The Journal of Immunology* **201**, 982-994 (2018).

16. Ru, J. *et al.* MiR-23a Facilitates the Replication of HSV-1 through the Suppression of Interferon Regulatory Factor 1. *PLOS ONE* **9**, e114021 (2014).

17. Xie, Y., He, S. & Wang, J. MicroRNA-373 facilitates HSV-1 replication through suppression of type I IFN response by targeting IRF1. *Biomedicine & Pharmacotherapy* **97**, 1409-1416 (2018).

18. Chen, Y. *et al.* HCV-Induced miR-21 Contributes to Evasion of Host Immune System by Targeting MyD88 and IRAK1. *PLOS Pathogens* **9**, e1003248 (2013).

19. Cheng, Y.Q. *et al.* Micro RNA‐155 regulates interferon‐γ production in natural killer cells via T im‐3 signalling in chronic hepatitis C virus infection. *Immunology* **145**, 485-497 (2015).

20. He, X., Teng, J., Cui, C., Li, D. & Wen, L. MicroRNA-182 inhibits HCMV replication through activation of type I IFN response by targeting FOXO3 in neural cells. *Experimental Cell Research* **369**, 197-207 (2018).

21. Li, S. *et al.* Micro RNA‐130a inhibits HCV replication by restoring the innate immune response. *Journal of viral hepatitis* **21**, 121-128 (2014).

22. Rastogi, M. & Singh, S.K. Modulation of Type-I Interferon Response by hsa-miR-374b-5p During Japanese Encephalitis Virus Infection in Human Microglial Cells. *Frontiers in Cellular and Infection Microbiology* **9** (2019).

23. Zhu, B. *et al.* MicroRNA-15b Modulates Japanese Encephalitis Virus–Mediated Inflammation via Targeting RNF125. *The Journal of Immunology* **195**, 2251-2262 (2015).

24. Thounaojam Menaka, C. *et al.* MicroRNA 155 Regulates Japanese Encephalitis Virus-Induced Inflammatory Response by Targeting Src Homology 2-Containing Inositol Phosphatase 1. *Journal of Virology* **88**, 4798-4810 (2014).

25. Zhu, X. *et al.* MicroRNA-30e* Suppresses Dengue Virus Replication by Promoting NF-κB–Dependent IFN Production. *PLOS Neglected Tropical Diseases* **8**, e3088 (2014).

26. Su, Y.C. *et al.* MicroRNA-155 inhibits dengue virus replication by inducing heme oxygenase-1-mediated antiviral interferon responses. *FASEB journal : official publication of the Federation of American Societies for Experimental Biology* **34**, 7283-7294 (2020).

27. Yu, K. *et al.* MicroRNA-548j inhibits type I interferon production by targeting ZBTB11 in patients with chronic hepatitis B. *Biochem Biophys Res Commun* **488**, 628-633 (2017).

28. Li, H. *et al.* Peste des Petits Ruminants Virus-Induced Novel MicroRNA miR-3 Contributes To Inhibit Type I IFN Production by Targeting IRAK1. *Journal of Virology* **95**, 10.1128/jvi.02045-02020 (2021).

29. Chang, X. *et al.* miR‐382‐5p promotes porcine reproductive and respiratory syndrome virus (PRRSV) replication by negatively regulating the induction of type I interferon. *The FASEB Journal* **34**, 4497-4511 (2020).

30. Zhang, Q. *et al.* MicroRNA-23 inhibits PRRSV replication by directly targeting PRRSV RNA and possibly by upregulating type I interferons. *Virology* **450-451**, 182-195 (2014).

31. Jia, X. *et al.* Cellular microRNA miR-26a suppresses replication of porcine reproductive and respiratory syndrome virus by activating innate antiviral immunity. *Scientific Reports* **5**, 10651 (2015).

32. Ren, T. *et al.* MicroRNA-4331-5p promotes FMDV replication through inhibiting interferon pathways in PK-15 cells. *Virus research* **286**, 198064 (2020).

33. Li, J. *et al.* MicroRNA-30a Modulates Type I Interferon Responses to Facilitate Coxsackievirus B3 Replication Via Targeting Tripartite Motif Protein 25. *Frontiers in immunology* **11** (2021).

34. Zheng, H.-q. *et al.* miR-615 facilitates porcine epidemic diarrhea virus replication by targeting IRAK1 to inhibit type III interferon expression. *Frontiers in Microbiology* **13** (2022).

35. Zheng, H. *et al.* in International Journal of Molecular Sciences, Vol. 19 (2018).

36. Ouyang, W., Wang, Y.-s., Du, X.-n., Liu, H.-j. & Zhang, H.-b. gga-miR-9* inhibits IFN production in antiviral innate immunity by targeting interferon regulatory factor 2 to promote IBDV replication. *Veterinary Microbiology* **178**, 41-49 (2015).

37. Fu, M. *et al.* MicroRNA gga-miR-130b Suppresses Infectious Bursal Disease Virus Replication via Targeting of the Viral Genome and Cellular Suppressors of Cytokine Signaling 5. *Journal of Virology* **92**, 10.1128/jvi.01646-01617 (2017).

38. Zhang, C. *et al.* MicroRNA miR-155 inhibits cyprinid herpesvirus 3 replication via regulating AMPK-MAVS-IFN axis. *Developmental & Comparative Immunology* **129**, 104335 (2022).

39. Blümke, J. *et al.* Identification and characterization of the anti-viral interferon lambda 3 as direct target of the Epstein-Barr virus microRNA-BART7-3p. *Oncoimmunology* **12**, 2284483 (2023).

40. Cox, J.E., McClure, L.V., Goga, A. & Sullivan, C.S. Pan-viral-microRNA screening identifies interferon inhibition as a common function of diverse viruses. *Proceedings of the National Academy of Sciences* **112**, 1856-1861 (2015).

41. Ramalingam, D. & Ziegelbauer, J.M. Viral microRNAs Target a Gene Network, Inhibit STAT Activation, and Suppress Interferon Responses. *Scientific Reports* **7**, 40813 (2017).

42. Bouvet, M. *et al.* Multiple Viral microRNAs Regulate Interferon Release and Signaling Early during Infection with Epstein-Barr Virus. *mBio* **12**, 10.1128/mbio.03440-03420 (2021).

43. Duan, Y. *et al.* Herpes Simplex Virus Type 1–Encoded miR-H2-3p Manipulates Cytosolic DNA–Stimulated Antiviral Innate Immune Response by Targeting DDX41. *Viruses* **11**, 756 (2019).

44. Bernard, M.A. *et al.* Novel HIV-1 MiRNAs Stimulate TNFα Release in Human Macrophages via TLR8 Signaling Pathway. *PLOS ONE* **9**, e106006 (2014).

45. Qian, Z. *et al.* Hepatitis E virus‐encoded microRNA promotes viral replication by inhibiting type I interferon. *The FASEB Journal* **36**, e22104 (2022).
